# Efficacy and Safety of Different Trapezium Implants for Trapeziometacarpal Joint Osteoarthritis: A Systematic Review and Meta-Analysis

**DOI:** 10.1177/15589447231183172

**Published:** 2023-07-02

**Authors:** Ishith Seth, Gabriella Bulloch, Nimish Seth, Quentin Fogg, David J. Hunter-Smith, Warren M. Rozen

**Affiliations:** 1 Monash University, Melbourne, VIC, Australia; 2 The University of Melbourne, VIC, Australia; 3 The Alfred Hospital, Melbourne, VIC, Australia

**Keywords:** trapezium implants, basal thumb arthritis, arthroplasty, trapeziometacarpal, arthritis

## Abstract

**Background:** The trapeziometacarpal joint (TMCJ) is the most common hand joint affected by osteoarthritis (OA), and trapezium implant arthroplasty is a potential treatment for recalcitrant OA. This meta-analysis aimed to investigate the efficacy and safety of various trapezium implants as an interventional option for TMCJ OA. **Methods:** Web of Science, PubMed, Scopus, Google Scholar, and Cochrane library databases were searched for relevant studies up to May 28, 2022. Preferred Reported Items for Systematic Review and Meta-Analysis guidelines were adhered to, and the protocol was registered in PROSPERO. The methodological quality was assessed by National Heart, Lung, and Blood Institute tools for observational studies and the Cochrane risk of bias tool. Subgroup analyses were performed on different replacement implants; the analysis was done using Open Meta-Analyst software and *P* values <.05 were considered statistically significant. **Results:** A total of 123 studies comprising 5752 patients were included. Total joint replacement (TJR) implants demonstrate greater significant improvements in visual analogue scale pain scores postoperatively. Interposition with partial trapezial resection implants were associated with highest grip strength and highest reduction in the Disabilities of the Arm, Shoulder, and Hand (DASH) score. Revision rates were highest in TJR (12.3%) and lowest in interposition with partial trapezial resection (6.2%). **Conclusion:** Total joint replacement and interposition with partial trapezial resection implants improve pain score, grip strength, and DASH scores more than other implant options. Future studies should focus on high-quality randomized clinical trials comparing different implants to accumulate higher quality evidence and more reliable conclusions.

## Introduction

The trapeziometacarpal joint (TMCJ) allows for the circumduction of the thumb necessary for grasping and pinching.^1^ Progressive age-related ligamentous laxity, trapezial joint surface hypoplasia, and abductor pollicis longus abnormalities,^2,3^ combined with degeneration of trapezium cartilage from load-bearing, make TMCJ extremely susceptible to osteoarthritis (OA).^4^ The TMCJ OA can precipitate weakness, reduced range of motion, and hand function deterioration, ultimately leading to reduced grip strength^5^ and affecting quality of life.^6^

Patients with intolerable pain and functional impairments refractory to conservative treatment are candidates for surgical intervention; however, optimal surgical methods and prosthesis for improving pain and functional outcomes are still controversial.^4,7^ Joint replacement and implants are a last resort for OA and include prosthetic interposition, total joint replacement (TJR), and hemiarthroplasty. Trapezium implants have varying degrees of success and popularity, but all carry high failure rates (17.2%-44.78%, 10-year failure rate) due to loosening and/or dislocation, persistent pain, infection/synovitis risk, and foreign body reactions.^4,7^ The theoretical advantages of using an implant to treat TMCJ OA include immediate thumb stability, restoring or enhancing joint biomechanics, preserving thumb length, patient satisfaction, and quicker recovery time, but which implant type would be the best choice is yet to be ascertained.^8^

This systematic review and meta-analysis aimed to summarize the relevant literature on TMCJ OA surgical implants to address the strengths and weaknesses of various implants and assist surgeons in choosing from the implant options available. Complications and failure rates, pain scores, functional assessments, and patient satisfaction with different trapezium implants were also explored.

## Materials and Methods

This meta-analysis was conducted using the *Cochrane Handbook for Systematic Reviews of Interventions*^9^ and Preferred Reported Items for Systematic Review and Meta-Analysis (PRISMA).^10^ The protocol was registered in Prospective Register of Systematic Reviews (PROSPERO; registration number: CRD42021271122).

### Literature Search

Web of Science, Scopus, PubMed, Google Scholar, and Cochrane Library databases were searched for relevant articles up to May 28, 2022. The search terms included “trapeziometacarpal” or “base of thumb” or “thumb” “CMC” or “CMCJ” or “TMCJ” or “TMC” and “implants” or “arthroplasty” and were modified according to the recommendations of each database. No age, gender, and population filters were imposed. Search terms are listed in Appendix 1. Duplicates were omitted using EndNote X9. Screening of titles and abstracts and then full texts was done by 2 authors (IS and GB); any discrepancies were resolved by the third author (NS). Manual search of references for included studies was also performed.

### Eligibility Criteria

The inclusion criteria were as follows: study designs including: (1) patients with TMCJ arthritis; (2) different types of trapezium implants such as TJR implants, hemiarthroplasty implants, interposition with partial trapezial resection implants, and interposition with total trapezial replacement implants; and (3) reported outcomes with one or more of the following: pain relief, Disabilities of the Arm, Shoulder, and Hand (DASH) scores, patient satisfaction, failure or revision rates, and complications. Original articles were eligible if they matched any of the first 4 levels of evidence classified in 2011 by the Oxford Centre for Evidence-Based Medicine (OCEBM).^11^ The studies were restricted to English language and a sample size of 20 patients or more.

Exclusion criteria were as follows: study designs including reviews, case reports or series, letter to the editor, conference abstracts, animal studies, experimental, or biomechanical studies; studies using nonimplant surgeries, medical treatment; studies in which full text was unavailable or with unextractable data; and studies not in English language.

### Data Extraction

The following data were extracted:

1. Study characteristics including sample size, level of evidence, surgical implant used, and follow-up.
2. Outcomes: the visual analogue scale (VAS) pain scores at rest, ranging from 0 to 10; incidence of implant failure or revision; complications; patient satisfaction; and DASH score. The DASH score ranges from 0 to 100. Post-treatment outcomes and changes from baseline values in the available studies were collected. In addition, different pain scales were standardized and converted to scales ranging from 0 to 10 similar to the VAS.
3. Domains for assessment of methodological quality.

### Risk of Bias Assessment

Methodological quality of the included observational and single-arm studies was assessed using the National Heart, Lung, and Blood Institute (NHLBI) tool.^12^ Questions about methodology were answered with “yes,” “no,” or “other.” The Cochrane risk of bias tool^13^ evaluated randomized controlled trials (RCTs) using the following domains: (1) randomization; (2) allocation; (3) blinding of patients and personnel; (4) blinding of outcome assessors; (5) incomplete outcome data; (6) selective reporting; and (7) any other sources of bias. Each domain is categorized as “low risk,” “high risk,” or “unclear risk” of bias.

### Data Synthesis and Analysis

In this meta-analysis, Open Meta-Analyst software (version 12.11.14) was used to pool estimates of the effects of the included study arms. Continuous data were exhibited as mean difference (MD) and 95% confidence interval (95% CI); dichotomous data were exhibited as incidence (%) and 95% CI. The DerSimonian random-effects method was used for the analysis. Subgroup analyses depending on the type of implant, TJR implants, hemiarthroplasty implants, interposition with partial trapezial resection implants, or interposition with total trapezial replacement implants were performed. Studies with substantial heterogeneity were investigated with chi-square (Q^2^) analysis and quantified by *I*-square (*I*^2^) tests. *I*^2^ value more than 50% and *P* value of Q^2^ < .1 was considered statistically significant. Significant heterogeneity was addressed by a random-effects model and tried to be resolved by sensitivity analysis, one study at a time.

## Results

### Search Results

**Figure 1.**
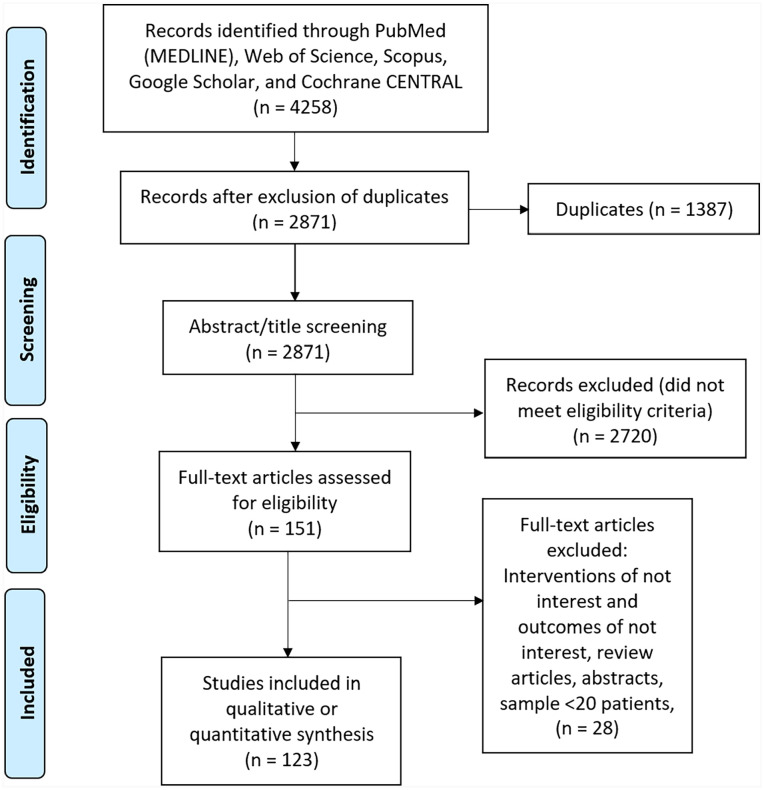
Preferred Reported Items for Systematic Review and Meta-Analysis flow diagram of the included studies.

A total of 4258 records were extracted in the initial search, and after the removal of duplicates, 2871 records were selected for titles and full-text screening. Ultimately, 123 studies, 3 RCTs, 80 cohort studies, and 40 single-arm studies, were included. (References for the included studies can be found in Appendix 6.) The study selection process is shown in Figure 1.

### Characteristics of the Included Studies

Of the included studies, there were 3167 TJRs, 422 hemiarthroplasties, 859 interpositions with partial trapezial resections, and 1304 interpositions with total trapezial replacements. The mean age range of enrolled patients was 40 to 72.5 years, and follow-up period ranged from 12 to 228 months. A summary of the design and baseline characteristics of enrolled patients is presented in Appendix 2.

On assessing the methodological quality of the included cohorts, 26 studies were of fair quality, and 54 studies were of poor quality (Appendices 3-5). Of the single-arm studies, 29 studies were of fair quality, and 11 studies were of poor quality. The 3 included RCTs were of overall high quality.

### Outcomes

#### Postoperative pain score

**Figure 2.**
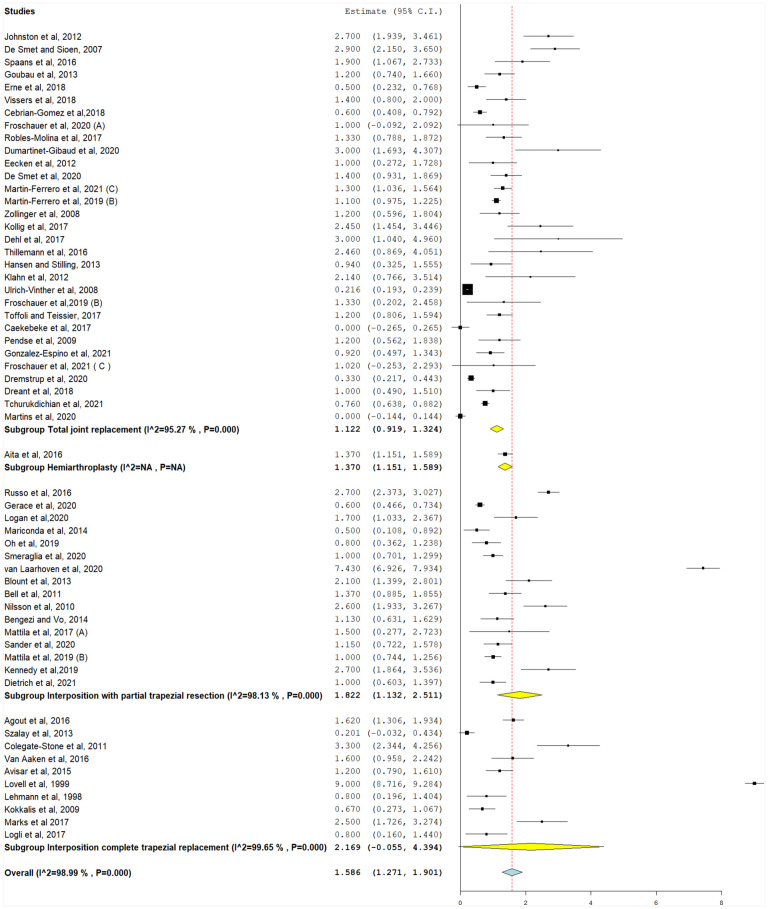
Change in pain score from baseline values (0-10), with the following subgroups: total joint replacement, interposition with partial trapezial resection, and interposition with total trapezial replacement. *Note.* CI = confidence interval.

Postoperative VAS scores of TJR, hemiarthroplasty, interposition with partial trapezial resection, and total trapezial replacement are reported in Figure 2. The average VAS pain score reduction (preoperative pain score subtracted from postoperative pain score) across implant types was 1.58 (95% CI, 1.271-1.901).

The postoperative pain score was between 0 and 10 and a higher number equates to greater pain. There was significant heterogeneity for TJR, interposition with partial trapezial resection, and interposition with total trapezial replacement studies (*I*^2^ = 95.2%, 98.1%, and 99.6%, respectively) (*P* < .001), as shown in Figure 2. Pooled heterogeneity was significant and could not be resolved (*I*^2^ = 98.9%, *P* < .001).

#### Change in pain score

**Figure 3.**
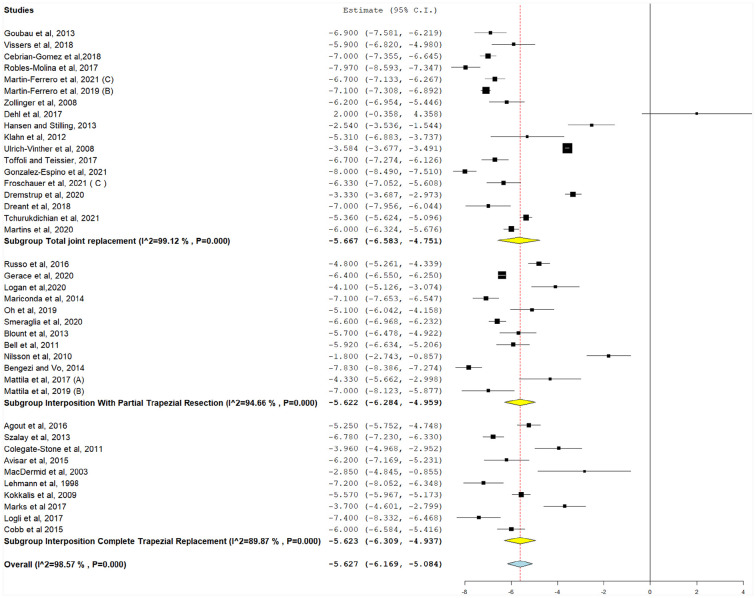
Postoperative pain scores (0-10), with the following subgroups: total joint replacement, hemiarthroplasty, interposition with partial trapezial resection, and interposition with total trapezial replacement. *Note.* CI = confidence interval.

For this analysis, VAS was calculated as MD by subtracting preoperative VAS pain score from postoperative VAS pain score. This was reported in 40 studies (Figure 3). For reporting of VAS pain score reductions, MD was highest in TJR (−5.66, 95% CI, −6.58 to −4.75), followed by interposition with partial trapezial resection (−5.62, 95% CI, −6.28 to −4.96) and interposition with total trapezial replacement (−5.62, 95% CI, −6.31 to −4.94) (*P* < .001). Different implants were associated with significant MDs in pain scores (−5.63, 95% CI, −6.17 to −5.08, *P* < .001).

Significant heterogeneity was detected in all studies after stratifying for implant types and could not be resolved (*P* < .001). Their pooled heterogeneity was also significant and could not be resolved (*I*^2^ = 98.5%, *P* < .001).

#### Patient satisfaction after treatment

**Figure 4.**
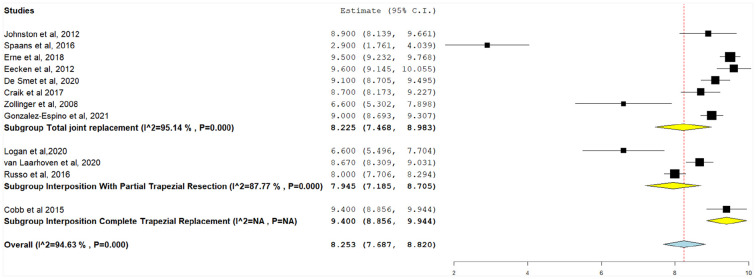
Postoperative patient satisfaction (1-10), with the following subgroups: total joint replacement, interposition with partial trapezial resection, and interposition with total trapezial replacement. *Note.* CI = confidence interval.

Patient satisfaction following treatment was assessed using the Likert scale (ranging from 0 to 10, higher number equates to greater satisfaction), and the pooled results ranged from 2.9 to 9.6. A total of 12 studies reported patient satisfaction following trapezium implant intervention (Figure 4). Using pooled data, patient satisfaction was highest in TJR (8.25, 95% CI, 7.47-8.98), followed by interposition with partial trapezium resection (7.94, 95% CI, 7.185-8.71). Both outcomes had significant unresolvable heterogeneity (*P* < .001). Only Cobb et al^14^ reported patient satisfaction for interposition with total trapezial replacement (9.40, 95% CI, 8.86-9.94). The overall patient satisfaction for all implants was 8.25 (95% CI, 7.69-8.82), with significant unresolvable heterogeneity (*I*^2^ = 94.6%, *P* < .001).

#### Postoperative grip strength

**Figure 5.**
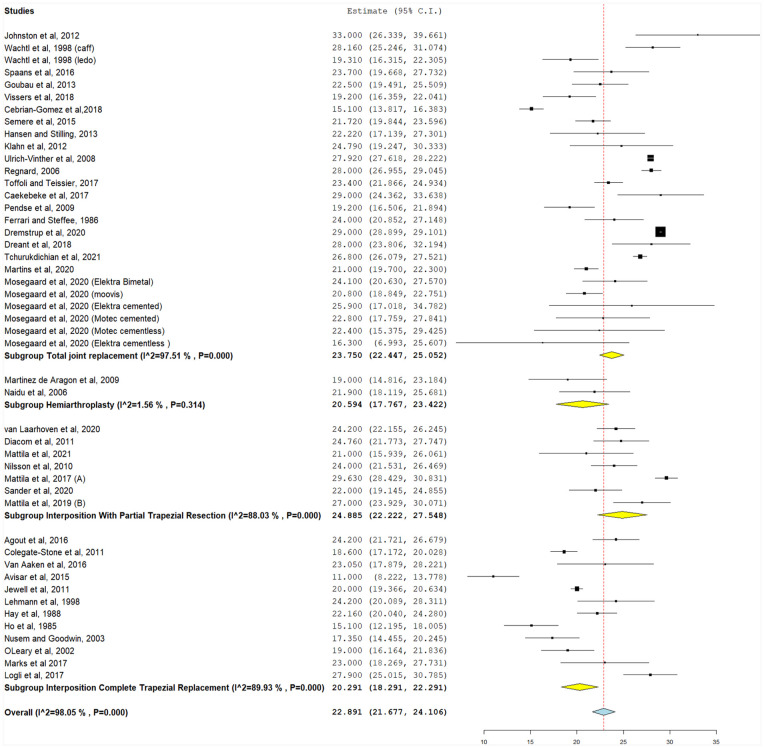
Change in grip strength from baseline values (kg), with the following subgroups: total joint replacement, hemiarthroplasty, interposition with partial trapezial resection, and interposition with total trapezial replacement. *Note.* CI = confidence interval.

**Figure 6.**
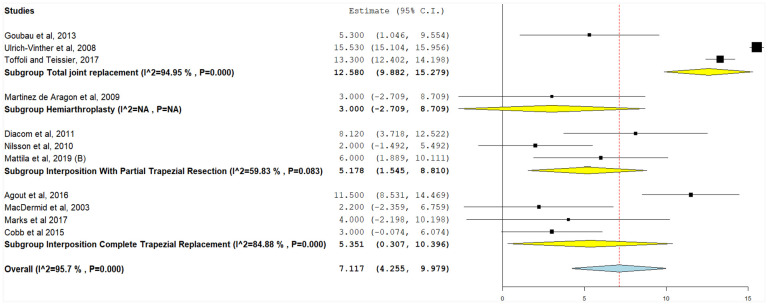
Postoperative grip strength (kg), with the following subgroups: total joint replacement, hemiarthroplasty, interposition with partial trapezial resection, and interposition with total trapezial replacement. *Note.* CI = confidence interval.

Grip strength was assessed using a hand dynamometer (Jamar, China) in some studies or a pinch gauge in 10 studies. A total of 47 implant arms assessed grip strength following trapezium implants (Figure 5). Postoperative grip strength was highest in interposition with partial trapezial resection (24.88 kg, 95% CI, 22.22-27.55), followed by TJR (23.75 kg, 95% CI, 22.45-25.05), hemiarthroplasty (20.59 kg, 95% CI, 17.77-23.42), and interposition with total trapezial replacement (20.29 kg, 95% CI, 18.29-22.29) (Figure 6). Inter-heterogeneity was 97.5%, 88.03%, and 89.93% for interposition with partial trapezial resection, TJR, and interposition with total trapezial replacement, respectively, and could not be resolved (*P* < .001). Hemiarthroplasty had low heterogeneity (*I*^2^ = 1.5%, *P* = .31), however only in 2 studies. Pooled grip strength for all implant operations was 22.89 kg (95% CI, 21.67-24.11), and significant heterogeneity could not be resolved (*I*^2^ = 98.05%, *P* < .001).

#### Change in grip strength

The difference between preoperative and postoperative grip strength was reported in 11 studies (Figure 6). The greatest improvements were observed in TJR (12.58 kg, 95% CI, 9.88-15.28, *P* < .001), followed by interposition with total trapezial replacement (5.35 kg, 95% CI, 0.31-10.37, *P* = .03), interposition with partial trapezial resection (5.18 kg, 95% CI, 1.55-8.81, *P* = .005), and hemiarthroplasty from a single study^15^ (3.00 kg, 95% CI, −2.709-8.709). There was significant heterogeneity in all studies that could not be resolved (*P* < .001). When implant types were pooled, significant grip strength improvements were observed (7.12 kg, 95% CI, 4.26-9.98, *P* < .001). Pooled heterogeneity was significant and could not be resolved (*I*^2^ = 95.7%, *P* < .001).

#### Postoperative DASH score

There were 64 implant arms (n = 2842) reporting DASH score after TJR (DASH = 23.27, 95% CI, 19.78-26.77), but heterogeneity was significant and could not be resolved (*I*^2^ = 98.7%, *P* < .001). A single study showed hemiarthroplasty resulted in the lowest DASH score (9.66, 95% CI, 5.86-13.46), followed by 15 studies reporting interposition with partial trapezial resection (18.49, 95% CI, 14.56-22.42) and 7 for total trapezial replacement (21.31, 95% CI, 15.28-27.33). Heterogeneity for interposition and total trapezial replacement was significant and could not be resolved (*P* < .001). Overall, the average DASH score after surgery was 21.72 (95% CI, 18.84-24.61), with significant pooled heterogeneity that could not be resolved (*I*^2^ = 99.16%, *P* < .001) (Supplementary Figure 1).

#### DASH score change

Improvements in DASH score were reported in 29 studies incorporating 1267 patients (Supplementary Figure 2). The greatest improvements were observed in interposition with partial trapezial resection (−38.68, 95% CI, −45.39 to 31.98; *P* < .001), followed by TJR (−33.09, 95% CI, −38.28 to −27.91; *P* < .001) and interposition with total trapezial replacement (−24.71, 95% CI, −45.851 to −3.58; *P* = .038). Significant heterogeneity was detected in all studies after stratifying for implant types and could not be resolved (*P* < .001). When all implant types were pooled, a significant reduction in DASH scores was observed (−34.15, 95% CI, −38.21 to −30.08, *P* < .001) and heterogeneity was significant without resolution (*I*^2^ = 97.72%, *P* < .001).

#### Implant failure

Implant failure was recorded when the implant was loose or dislocated and was reported in 130 studies (Supplementary Figure 3). Implant failure was highest in TJR (12.3%), followed by hemiarthroplasty (10.2%), interposition with total trapezial replacement (7.9%), and interposition with partial trapezial resection (6.2%). Significant heterogeneity was detected in all studies after stratifying for implant types and could not be resolved (*P* < .001). After pooling implant types, incidence of implant failure was 9.8% (n = 744/6657), and heterogeneity could not be resolved (*I*^2^ = 81.35%, *P* < .001).

#### Complications

Postoperative complications were reported in 121 studies (Supplementary Figure 4) and included dislocation, periprosthetic ossification, fracture, infection, and revision/failure. These were highest after interposition with total trapezial replacement (26.7%), followed by TJR (21.7%), partial trapezial resections (17.6%), and hemiarthroplasty (13.3%) in all pooled studies. Significant heterogeneity was detected in all studies after stratifying for implant types and could not be resolved (*P* < .001). After pooling studies for all complications, the incidence of complications was 21.5% (n = 1252/6374), and heterogeneity could not be resolved (*I*^2^ = 92.6%, *P* < .001).

## Discussion

This study investigated the efficacy of trapezium implants for the treatment of TMCJ OA and found that TJR not only significantly improved postoperative pain scores and grip strength but also had the highest implant failure rate. Meanwhile, higher grip strength and DASH scores were reported for interposition with partial trapezium resection surgeries. Significant heterogeneity was observed throughout the analyses, and half of the included studies were of poor quality according to the NHLBI criteria. Considering these findings, a few major discussion points should be addressed.

Total joint replacement yielded the greatest functional improvements following TMCJ OA; however, it also had the highest risk of implant failure. These findings are different from Wilkens et al,^16^ who compared nonoperative treatment with arthroscopic-assisted techniques across 10 studies and found no differences between arthroscopic-assisted techniques; however, the impressive cohort and number of studies in this analysis likely reflect real-world outcomes more precisely. The findings favoring TJR may be due to surgeries often providing hand therapy and postoperative rehabilitation more than other surgical interventions, which may confer patient satisfaction, grip strength, and lower VAS. Grip strength has been linked with strength of other muscle groups and is a good predictor of overall strength.^17^ Therefore, the significant grip strength improvement noted in TJR could improve overall strength and lower the risk of physical disability and falls compared with other interventions.^17^ In addition, significant lower VAS score noted in TJR postoperatively would be conducive to thumb use, thereby facilitating similar favorable outcomes. Despite this, TJR carried higher rates of implant failure and the second highest rate of complications inferring the surgery is still not optimized. A recent review by Bæk Hansen^18^ postulates that the reliability of joint replacements has grown in the past 10 years; however, surgeon-dependent factors, such as soft tissue release and the avoidance of removing excessive bony material, and patient factors, such as heavy or repetitive manual work postsurgery, likely also bear weight on complications and implant failure. Large cohorts using newer stem and cup designs have more recently shown 10-year survival is around 85% to 95%, although a large number of included studies in our meta-analysis have likely prevented this small proportion from being recognized.

Interposition with partial trapezial resection had inferior but comparable results to total TMCJ replacement with the highest improvement in DASH scores, second highest reduction in VAS score, and second highest patient satisfaction. In contrast to total TMCJ replacement, interposition with partial trapezial resection was associated with the lowest failure rate and second lowest incidence of complications, but this is inconsistent with a previous systematic review by Ganhewa et al^7^ where it had the highest rates of failure. This controversy could be attributed to using the linear estimation of failure rates that estimated the failure rate over time and did not account for actual failure rates. Overall, this study supports the use of this technique as a low-risk, high-return surgery for the treatment of TMCJ OA.

Hemiarthroplasty and interposition with total trapezial replacement were associated with poorer results which may be attributed to the sheer insufficiency of studies reporting the outcomes we addressed. For example, with hemiarthroplasty, only one study reported VAS scores, satisfaction, and DASH scores, which limits a pooled analysis and accurate comparison with other surgery types. In studies where there was more than one available study for analysis, hemiarthroplasty was associated with low revision and complication rates, which may confer to its short surgery times and minimal blood loss.^19^ Similarly, interposition with total trapezial replacement was associated with the poorest improvements in VAS scores and DASH scores, although its comparatively higher number of studies confers this to be a true association.

Currently, simple trapeziectomy (excision of the trapezium bone) and trapeziectomy with ligament reconstruction and tendon interposition (LRTI) are the most popular surgeries for managing TMCJ OA. A 2021 meta-analysis compared the outcomes of joint replacements with trapeziectomy and LRTI, showing more favorable outcomes for DASH and opposition in implant groups; however, complications were also higher.^20^ This is corroborated by the systematic review of Ganhewa et al showing that the overall failure rates in trapeziectomy alone or with LRTI were lower than the failure rates of all implant groups due to aseptic loosening, dislocation, and persisting pain caused by many implant techniques. A previous Cochrane review stated no technique overall was superior for pain and physical function when comparing simple trapeziectomy, trapeziectomy with LRTI, and some implant surgeries; however, Swanson implant and GraftJacket allograft were inferior to simple trapeziectomy and trapeziectomy with LRTI for improving physical performance, lowering pain and adverse event rates, and ensuring treatment satisfaction. It seems that the major shortfalls of implants are their complication and failure rates, which may be due to their lack of personalization and fit an individual’s anatomy. Considering the rise of 3D implanted joints, this approach should be considered by future surgeons.

This systematic review combined single-arm meta-analysis on this topic to compare the efficacy of implant types. Our study included 123 studies with an impressive sample size (n = 5752). Despite this, some limitations should be acknowledged. First, unresolvable heterogeneity was present among study arms in each category, even after random-effects model analysis and leave-one-out analysis. Therefore, these are likely from the various surgical approaches, biomaterials of implants, and follow-up durations. Second, surgeon experience is an unavoidable factor that inevitably lends itself to complications that affect functional outcomes and efficacy. Third, some studies had follow-up duration of less than 2 years which is insufficient for evaluating long-term safety and efficacy of treatments. Last, we could not find enough studies in the literature that report outcomes for interposition without trapezial resection, which prevents its efficacy from being assessed. Future randomized clinical trials comparing implant arthroplasty with non-alloplastic surgical options are needed.

In conclusion, this meta-analysis suggests that TJR and interposition with partial trapezial resection are surgeries that hold superior efficacy to other implant options for the treatment of TMCJ OA. Significant heterogeneity and a low number of studies available for assessing the efficacy of hemiarthroplasty may limit the generalizability of these results. Future high-quality randomized clinical trials comparing different implant options should refine these findings and provide higher evidence for informing clinical practice.

## Supplemental Material

sj-docx-1-han-10.1177_15589447231183172 – Supplemental material for Efficacy and Safety of Different Trapezium Implants for Trapeziometacarpal Joint Osteoarthritis: A Systematic Review and Meta-Analysis Supplemental material, sj-docx-1-han-10.1177_15589447231183172 for Efficacy and Safety of Different Trapezium Implants for Trapeziometacarpal Joint Osteoarthritis: A Systematic Review and Meta-Analysis by Ishith Seth, Gabriella Bulloch, Nimish Seth, Quentin Fogg, David J. Hunter-Smith and Warren M. Rozen in HAND

sj-docx-2-han-10.1177_15589447231183172 – Supplemental material for Efficacy and Safety of Different Trapezium Implants for Trapeziometacarpal Joint Osteoarthritis: A Systematic Review and Meta-Analysis Supplemental material, sj-docx-2-han-10.1177_15589447231183172 for Efficacy and Safety of Different Trapezium Implants for Trapeziometacarpal Joint Osteoarthritis: A Systematic Review and Meta-Analysis by Ishith Seth, Gabriella Bulloch, Nimish Seth, Quentin Fogg, David J. Hunter-Smith and Warren M. Rozen in HAND

sj-docx-3-han-10.1177_15589447231183172 – Supplemental material for Efficacy and Safety of Different Trapezium Implants for Trapeziometacarpal Joint Osteoarthritis: A Systematic Review and Meta-Analysis Supplemental material, sj-docx-3-han-10.1177_15589447231183172 for Efficacy and Safety of Different Trapezium Implants for Trapeziometacarpal Joint Osteoarthritis: A Systematic Review and Meta-Analysis by Ishith Seth, Gabriella Bulloch, Nimish Seth, Quentin Fogg, David J. Hunter-Smith and Warren M. Rozen in HAND

sj-docx-4-han-10.1177_15589447231183172 – Supplemental material for Efficacy and Safety of Different Trapezium Implants for Trapeziometacarpal Joint Osteoarthritis: A Systematic Review and Meta-Analysis Supplemental material, sj-docx-4-han-10.1177_15589447231183172 for Efficacy and Safety of Different Trapezium Implants for Trapeziometacarpal Joint Osteoarthritis: A Systematic Review and Meta-Analysis by Ishith Seth, Gabriella Bulloch, Nimish Seth, Quentin Fogg, David J. Hunter-Smith and Warren M. Rozen in HAND

sj-docx-5-han-10.1177_15589447231183172 – Supplemental material for Efficacy and Safety of Different Trapezium Implants for Trapeziometacarpal Joint Osteoarthritis: A Systematic Review and Meta-Analysis Supplemental material, sj-docx-5-han-10.1177_15589447231183172 for Efficacy and Safety of Different Trapezium Implants for Trapeziometacarpal Joint Osteoarthritis: A Systematic Review and Meta-Analysis by Ishith Seth, Gabriella Bulloch, Nimish Seth, Quentin Fogg, David J. Hunter-Smith and Warren M. Rozen in HAND

sj-docx-6-han-10.1177_15589447231183172 – Supplemental material for Efficacy and Safety of Different Trapezium Implants for Trapeziometacarpal Joint Osteoarthritis: A Systematic Review and Meta-Analysis Supplemental material, sj-docx-6-han-10.1177_15589447231183172 for Efficacy and Safety of Different Trapezium Implants for Trapeziometacarpal Joint Osteoarthritis: A Systematic Review and Meta-Analysis by Ishith Seth, Gabriella Bulloch, Nimish Seth, Quentin Fogg, David J. Hunter-Smith and Warren M. Rozen in HAND

sj-tiff-10-han-10.1177_15589447231183172 – Supplemental material for Efficacy and Safety of Different Trapezium Implants for Trapeziometacarpal Joint Osteoarthritis: A Systematic Review and Meta-Analysis Supplemental material, sj-tiff-10-han-10.1177_15589447231183172 for Efficacy and Safety of Different Trapezium Implants for Trapeziometacarpal Joint Osteoarthritis: A Systematic Review and Meta-Analysis by Ishith Seth, Gabriella Bulloch, Nimish Seth, Quentin Fogg, David J. Hunter-Smith and Warren M. Rozen in HAND

sj-tiff-7-han-10.1177_15589447231183172 – Supplemental material for Efficacy and Safety of Different Trapezium Implants for Trapeziometacarpal Joint Osteoarthritis: A Systematic Review and Meta-Analysis Supplemental material, sj-tiff-7-han-10.1177_15589447231183172 for Efficacy and Safety of Different Trapezium Implants for Trapeziometacarpal Joint Osteoarthritis: A Systematic Review and Meta-Analysis by Ishith Seth, Gabriella Bulloch, Nimish Seth, Quentin Fogg, David J. Hunter-Smith and Warren M. Rozen in HAND

sj-tiff-8-han-10.1177_15589447231183172 – Supplemental material for Efficacy and Safety of Different Trapezium Implants for Trapeziometacarpal Joint Osteoarthritis: A Systematic Review and Meta-Analysis Supplemental material, sj-tiff-8-han-10.1177_15589447231183172 for Efficacy and Safety of Different Trapezium Implants for Trapeziometacarpal Joint Osteoarthritis: A Systematic Review and Meta-Analysis by Ishith Seth, Gabriella Bulloch, Nimish Seth, Quentin Fogg, David J. Hunter-Smith and Warren M. Rozen in HAND

sj-tiff-9-han-10.1177_15589447231183172 – Supplemental material for Efficacy and Safety of Different Trapezium Implants for Trapeziometacarpal Joint Osteoarthritis: A Systematic Review and Meta-Analysis Supplemental material, sj-tiff-9-han-10.1177_15589447231183172 for Efficacy and Safety of Different Trapezium Implants for Trapeziometacarpal Joint Osteoarthritis: A Systematic Review and Meta-Analysis by Ishith Seth, Gabriella Bulloch, Nimish Seth, Quentin Fogg, David J. Hunter-Smith and Warren M. Rozen in HAND
