# Supplementary material for: Efficacy and Safety of Different Trapezium Implants for Trapeziometacarpal Joint Osteoarthritis: A Systematic Review and Meta-Analysis
Source: Hand (N Y). 2023 Jul 2;19(8):1242–51. doi: 10.1177/15589447231183172 (PMC11612267; doi:10.1177/15589447231183172)
Supplement: sj-docx-1-han-10.1177_15589447231183172 – Supplemental material for Efficacy and Safety of Different Trapezium Implants for Trapeziometacarpal Joint Osteoarthritis: A Systematic Review and Meta-Analysis [file sj-docx-1-han-10.1177_15589447231183172.docx]

**Search strategy**

(Trapeziometacarpal OR trapezio-metacarpal OR TMC OR trapezial-metacarpal OR trapezialmetacarpal OR thumb base OR base of thumb OR base of the thumb OR Rhizarthrosis OR first Carpometacarpal OR thumb Carpometacarpal OR first CMC OR CMC1 OR CMC 1 OR CMCJ OR thumb CMC OR first carpo-metacarpal OR Basilar thumb OR Basilar joint OR Basal joint OR basal thumb OR trapezium OR Trapezial OR carpal adj metacarpal OR thumb*)

AND

(implant OR total Joint replacement OR hemiarthroplasty OR interposition with partial trapezium* resection OR interposition with total trapezi* OR Interposition without trapezi* resection OR De la Caffiniere OR Caffinière OR Ivory OR total endoprosthesis OR ARPE OR Roseland OR Moje Acamo CMC OR Nahigian OR Rubis II OR Motec OR Metal on Metal OR MoM OR Polyethylene cemented OR PE cemented OR MAIA OR Elektra OR GUEPAR OR Moovis OR Avanta OR Braun-Cutter OR cementless OR non-cemented OR uncemented OR Ledoux OR Cemented metal and plastic OR Mayo OR Steffee OR Bichat OR Pyrohemisphere OR PyroSphere OR NuGrip OR BioPro OR Swanson OR Silicone OR Pyrocardan OR AREX OR Polylactic acid OR PLLA OR Cartiva Polyvinyl acid OR PVA OR Artelon OR spacer OR PyroDisk OR Pyrocarbon OR Pyrolytic Carbon OR Orthosphere OR Button OR Silicone-Rubber OR Ashworth OR Kessler OR Proplast OR Pi2 OR Tie-in OR Polyethylene-mesh OR polyethylene mesh implant OR Gelfoam OR Helal OR Gore-Tex OR Niebauer OR Tecoflex OR Polyurethane OR Eaton OR GraftJacket OR Graft Jacket OR Articulinx OR Permacol)

**Google Scholar, Scopus, and Web of Science:**

(Trapeziometacarpal OR "trapezio-metacarpal" OR TMC OR "trapezial-metacarpal" OR trapezialmetacarpal OR "thumb base" OR "base of thumb" OR "base of the thumb" OR Rhizarthrosis OR "first Carpometacarpal" OR "thumb Carpometacarpal" OR "first CMC" OR CMC1 OR "CMC 1" OR CMCJ OR "thumb CMC" OR "first carpo-metacarpal" OR "Basilar thumb" OR "Basilar joint" OR "Basal joint" OR "basal thumb" OR trapezium OR Trapezial OR "carpal adj metacarpal" OR thumb*)

AND

(implant OR "total Joint replacement" OR hemiarthroplasty OR "interposition with partial trapezi*" OR "interposition with total trapezi*" OR "Interposition without trapezi*" OR "De la Caffiniere" OR Caffinière OR Ivory OR "total endoprosthesis" OR ARPE OR Roseland OR "Moje Acamo" OR Nahigian OR "Rubis II" OR Motec OR "Metal on Metal" OR MoM OR "Polyethylene cemented" OR "PE cemented" OR MAIA OR Elektra OR GUEPAR OR Moovis OR Avanta OR "Braun-Cutter" OR cementless OR "non-cemented" OR uncemented OR Ledoux OR "Cemented metal and plastic" OR Mayo OR Steffee OR Bichat OR Pyrohemisphere OR PyroSphere OR NuGrip OR BioPro OR Swanson OR Silicone OR Pyrocardan OR AREX OR "Polylactic acid" OR PLLA OR "Cartiva Polyvinyl acid" OR PVA OR Artelon OR spacer OR PyroDisk OR Pyrocarbon OR "Pyrolytic Carbon" OR Orthosphere OR Button OR "Silicone-Rubber" OR Ashworth OR Kessler OR Proplast OR Pi2 OR "Tie-in" OR "Polyethylene-mesh" OR "polyethylene mesh implant" OR Gelfoam OR Helal OR "Gore-Tex" OR Niebauer OR Tecoflex OR Polyurethane OR Eaton OR GraftJacket OR "Graft Jacket" OR Articulinx OR Permacol)

***********************************************************

**PubMed:**

**Number of results:** 727

**Web of Science:**

**Number of results:** 1270

**Cochrane Library:**

**Number of results:** 209

**Scopus**:

**Number of results:** 1029

**Google Scholar:**

**Number of results:** 1023

***********************************************************

**Total number:** 4258

**Duplicates:** 1387

**Total after duplicates removed:** 2871

**Searching databases date:** 28/5/2022

***References from 41 – 143 can be found in Appedix 6**
