# Supplementary material for: Efficacy and Safety of Different Trapezium Implants for Trapeziometacarpal Joint Osteoarthritis: A Systematic Review and Meta-Analysis
Source: Hand (N Y). 2023 Jul 2;19(8):1242–51. doi: 10.1177/15589447231183172 (PMC11612267; doi:10.1177/15589447231183172)
Supplement: sj-docx-2-han-10.1177_15589447231183172 – Supplemental material for Efficacy and Safety of Different Trapezium Implants for Trapeziometacarpal Joint Osteoarthritis: A Systematic Review and Meta-Analysis [file sj-docx-2-han-10.1177_15589447231183172.docx]

**Supplementary Table 1:** summary of the included studies

| **Study ID** | **Intervention (implant or control group)** | **Implant category** | **Sample size** | **Level of evidence** | **Mean age, Y** | **Mean follow up, M** | **Inclusion criteria** | **Outcomes** | **Conclusion** |
| --- | --- | --- | --- | --- | --- | --- | --- | --- | --- |
| **Johnston et al, 2012** | De la Caffinière prosthesis | Total joint replacement | 26 | IV | 57 | 228 | 1) Patients had a de la Caffinière prosthesis implanted between 1980 and 1989 | 1) Patient satisfaction 2) Pain 3) Grip strength 4) The DASH score 5) VAS score | “All such prostheses should remain under long-term surveillance following implantation and we would support the construction of a joint registry to record implant fate & revision rates.” |
| **De Smet and Sioen, 2007** | De la Caffinière prosthesis | Total joint replacement | 40 | IV | 54 | 26 | 1) All patients consulting for painful primary  osteoarthritis of the CMC-joint of the thumb not responding to conservative treatment | 1) The DASH score 2) ROM 3) Key pinch force 4) Patient satisfaction | “Although the outcome of the three procedures was not significantly different, trapeziectomy scored the  lowest in all parameters when compared with the LRTI patients. Arthroplasties were better but the high incidence of loosening is frightening for the future.” |
|  | Trapeziectomy |  | 22 |  | 61.5 | 34 |  |  |  |
|  | Trapeziectomy with LRTI |  | 34 |  | 58 | 26 |  |  |  |
| **Van Cappelle et al, 1999** | De la Caffinière prosthesis | Total joint replacement | 63 | IV | 62 | 102 | 1) First generation de la Caffinie`re prostheses were implanted into the hands | 1) Radiographic results 2) ROM 3) Pain 4) Power Grip | “If failure occurs, revision is preferred over arthrodesis or resection. When the feasibility of a revision is doubtful, a primary salvage procedure should be chosen.” |
| **Chakrabarti et al, 1997** | De la Caffinière prosthesis | Total joint replacement | 71 | IV | 57 | 132 | 1) Total joint replacements were implanted | 1) Revision rate 2) Complications 3) Patient satisfaction 4) Radiographic assessment | “We support the use of this implant for degenerative osteoarthritis, but caution against its use in men under 65 years” |
| **Sondergaard et al, 1991** | De la Caffinière prosthesis | Total joint replacement | 23 | IV | 60 | 108 | 1) Patients with Caffiniere prostheses in the trapezia-metacarpal joint. | 1) ROM 2) Radiographic assessment | “Replacement of loose components seems to be effective in case of aseptic loosening. If revision fails, we believe that the spectrum of other methods (arthrodesis or  resection with or without interposition) will offer a  satisfactory salvage procedures (Caffiniere and Rothe,1985)” |
| **Boeckstyns et al, 1989** | De la Caffinière prosthesis | Total joint replacement | 28 | IV | 62 | 48 | 1) Patients were treated for pain in the trapeziometacarpal joint. 2) In all cases a Caffiniere prosthesis was used | 1) Pain 2) Strength grip pinch 3) Complications 4) Radiographic results | “We believe that arthrodesis or interposition arthroplasty will be viable salvage procedures.” |
| **August et al, 1984** | De la Caffinière prosthesis | Total joint replacement | 20 | IV | 57 | 15.2 | 1) Patients with De La Caffiniere trapezio-metacarpal prostheses  inserted | 1) Pain 2) Strength grip pinch 3) Complications 4) Radiographic results | “In conclusion we feel that we have shown that there is an unacceptable rate of cup loosening which will probably not be altered until the cup fixation is altered. These  prostheses should be used for highly selective indications until it has been proven to be acceptable in  the long term.” |
| **De la Caffinière and Aucouturier, 1979** | De la Caffinière prosthesis | Total joint replacement | 29 | IV | 59 | 24 | 1) Patients with total trapezio-metacarpal prostheses | 1) Pain 2) Stability  3) Mobility | “The substitution of trapezio-metacarpal joint by total prosthesis provided us on the whole with good functional results; the follow up allows us now to state that after one year the good results are maintained. The first indication is carpometacarpal arthrosis provided that the trapezium has sufficient volume to allow drilling without fracture”. |
| **Wachtl et al, 1998** | De la Caffinière prosthesis | Total joint replacement | 41 | III | 61 | 63.5 | 1) Patients with primary osteoarthritis | 1) Complications 2) Revision  3) ROM 4) Strength 5) Radiographic assessment | “Both prostheses behaved similarly, and it is clear hat a constrained ball-and-socket prosthesis is not suitable for the trapeziometacarpal joint.” |
|  | Ledoux prosthesis |  | 43 |  |  | 25.3 |  |  |  |
| **Spaans et al, 2016** | Ivory TMC prosthesis | Total joint replacement | 20 | IV | 60 | 37 | 1) Patients with Ivory prostheses were placed | 1) DASH 2) ROM 3) VAS 4) MHQ | “Ivory prosthesis TMC arthroplasty can achieve good results in patients  with symptomatic isolated TMC OA. In this series, however, revision surgery was required in 3 of 20 cases.” |
| **Goubau et al, 2013** | Ivory TMC prosthesis | Total joint replacement | 22 | IV | 66 | 67 | 1) Patients Between 2005 and 2006, the Ivory prosthesis was  inserted with isolated primary TM degenerative osteoarthritis that had failed to respond  to conservative management. 2) None had a history of previous TM trauma, and none had undergone previous thumb surgery or other related treatment | 1) Strength 2) DASH 3) Pain 4) Radiographic assessment | “Our medium-term follow-up results (> 5 years) with a 95% survival rate indicate that the Ivory arthroplasty appears to be an effective surgical option for advanced TM osteoarthritis for patients in whom all conservative measures have failed. We strongly believe that future long-term studies in excess of 10 years post-operatively are essential before full validation of the efficacy of this implant can be realized.” |
|  |  |  |  |  |  |  |  |  |  |
| **Erne et al, 2018** | Ivory TMC prosthesis | Total joint replacement | 30 | III | 56.2 | 42 | 1) Patients with symptomatic osteoarthritis of the carpometacarpal I joint stage III according to the Eaton-Littler classification. | 1) DASH Score 2) Pain 3) Pinch force 4) VAS | “Both techniques resulted in improved function of the operated  hand and a clear reduction of symptoms. However, the implantation of a total endoprosthesis seems to have advantages, given a significantly better DASH score and a significantly shorter time until resolution of symptoms.” |
|  | Resection group |  | 25 |  | 54.3 | 36 |  |  |  |
| **Tchurukdichian et al, 2020** | Ivory TMC prosthesis | Total joint replacement | 95 | II | 61 | 120 | 1) The presence of pain at rest or affecting daily living activities, despite at least 3 months of conservative treatment. The indication for surgery was based on clinical features  rather than radiological appearances. | 1) Pain relief 2) Satisfaction 3) Key pinch 4) VAS 5) Survival rate 6) DASH 7) Radiographic assessment | “In conclusion, the long-term results with the IVORY prosthesis  are very satisfactory in terms of pain relief, function and survival.” |
| **Vissers et al, 2018** | Ivory TMC prosthesis | Total joint replacement | 24 | II | 71 | 130 | 1) Patients who were treated with an Ivory arthroplasty between 2005 and 2007 | 1) DASH 2) VAS 3) Survival rate | “Long-term results suggest the Ivory arthroplasty to be a reliable treatment for trapeziometacarpal osteoarthritis since it improves overall function and reduces pain up to 10 years postoperatively. However, revision within 10 years after surgery was needed in four of 26 cases” |
| **Cebrian-Gomez et al,2018** | Ivory TMC prosthesis | Total joint replacement | 84 | II | 60.4 | 45.6 | 1) The indications for LRTI and prosthesis were age over 50 years, radiographic evidence of advanced TMC arthritis failure and failed non-operative treatment, including steroid injections  and physical therapy for a minimum of 6 to 12 weeks | 1) DASH 2) Strength 3) VAS | “We conclude that trapeziometacarpal prosthesis provides better mid-term results in terms of function compared with ligament reconstruction and tendon interposition for patients with Stages 2 and 3 osteoarthritis of the trapeziometacarpal joint.” |
|  | LTRI |  | 62 |  | 60.9 | 43.2 |  |  |  |
| **Froschauer et al, 2020** | Ivory TMC prosthesis | Total joint replacement | 29 | III | 54.4 | 54 | 1) Patients who underwent either RA or Ivory prosthesis implantation for primary CMC-I osteoarthritis at our institution between January 2011 and December 2015 | 1) DASH 2) Strength 3) VAS 4) ROM 5) Satisfaction 6) Complications | “As the high complication rate is compensated by a better functional outcome (enhanced range of motion and strength), we believe that prosthesis implantation can be a reasonable treatment option for carpo-meta‑carpal‑I osteoarthritis in a particular patient group.” |
|  | Resection arthroplasty |  | 37 |  | 60.9 | 49.2 |  |  |  |
| **Robles-Molina et al, 2017,** | ARPE TMC prosthesis | Total joint replacement | 31 | III | 56.37 | 56 | 1) Patients with Eaton stage III carpometacarpal osteoarthritis of the thumb undergoing surgery between January 2006 and December 2011. | 1) DASH score  2)VAS score | “Ball and socket arthroplasty with Arpe prosthesis can be considered in selected patients in Eaton stage III because the benefits of improving pinch strength and  preventing hyperextension deformity outweigh the risk of dislocation, which can be avoided by a ligament reconstruction and tendon interposition procedure.” |
|  | Control with LTRI |  | 34 |  | 60.48 | 59 |  |  |  |
| **Eecken et al, 2012** | ARPE TMC prosthesis | Total joint replacement | 41 | IV | 55 | 72 | 1) Patients were operated on with a non-cemented total joint prosthesis (Arpe, Biomed). | 1) DASH 2) VAS | “To conclude, good subjective results and survival rates were obtained with the Arpe prosthesis at midterm, but longer-term survival rate is likely to decrease.” |
| **Martin-Ferrero, 2014 (A)** | ARPE TMC prosthesis | Total joint replacement | 60 | IV | 58 | 120 | 1) Patients for carpometacarpal joint osteoarthritis using the cementless hydroxyapatite (HA)-coated unconstrained ARPE implant. | 1) ROM 2) VAS score 3) Complications | “This study demonstrates that the ARPE thumb CMC joint prosthesis is a reliable option for patients with thumb CMC arthritis of Stage III and for some patients of Stage IV of the Eaton classification in patients with medium–high level of functional demand.” |
| **Martin-Ferrero et al, 2021 (C)** | ARPE TMC prosthesis | Total joint replacement | 60 | IV | 72.5 | 120 | 1) Patients must have osteoarthritis of Eaton’s grade II and III and some grade IV,  2) Be aged over 65 years, and may engage in light or medium but not excessively heavy manual tasks | 1) Radiological assessment 2) DASH 3) VAS 4) Kapandji score | “This series of patients with long follow-up has demonstrated that Arpe® prosthesis is a long lasting, effective and reliable alternative for surgical treatment of TMCOA in patients over 65 years of age, if it is performed with the criteria of surgical indication and surgical technique described throughout the study.” |
| **Dumartinet-Gibaud et al, 2020** | ARPE TMC prosthesis | Total joint replacement | 63 | IV | 59 | 120 | 1) Patients operated  between January 1994 and December 2007  2) Indication for  operation was TMC osteoarthritis stage III or IV according to the Dell classification  that had not responded to conservative treatment over at least 6 months. | 1) Survival rate  2) Complications | “We conclude that the implant survival declines progressively in the long run, with a survival  rate of 80% after 15 years of follow-up and a further decline thereafter. We also found that this surgery was difficult to master. We advise selecting this implant for thumb trapeziometacarpal osteoarthritis with caution.” |
| **De Smet et al, 2020** | ARPE TMC prosthesis | Total joint replacement | 57 | IV | 57 | 120 | 1) Patients were operated on with a total joint prosthesis (Arpe, Biomet, Warsaw, IN) for painful trapeziometacarpal osteoarthritis. | 1) Survival rate 2) DASH 3) Satisfaction 4) Complications 5) Pain | “It can be concluded that subjective outcomes  of patients with the Arpe prosthesis after a mean follow-up of ten years are good as long as no revision are needed. Patients should be informed that the risk of revision surgery after ten years might be as high as 20%” |
| **Craik et al 2017** | ARPE TMC prosthesis | Total joint replacement | 83 | III | 65 | 24 | 1) Patients treated with either trapeziectomy or ARPE thumb CMCJ arthroplasty | 1) Functional and 2) Satisfaction outcomes scores  3) The Disabilities of the Arm, Shoulder, and Hand questionnaire (DASH) | “Both trapeziectomy and the ARPE CMCJ arthroplasty are effective treatment options for thumb CMCJ osteoarthritis. Arthroplasty may offer potential advantages in terms of post-operative function and patient satisfaction. However the risk of complications and requirement for further surgery is greater and must be carefully considered during patient selection and preoperative counselling” |
|  | Trapeziectomy |  | 46 |  | 69 | 41 |  |  |  |
| **Martin-Ferrero et al, 2019 (B)** | ARPE TMC prosthesis | Total joint replacement | 228 | II | 59 | 120 | 1) Patients with CMC joint OA degree II and III of Eaton and Littler  and some degree IV,  2) Patient’s age  between 50 and 70 years,  3) Patients with small or medium demands and medium-to-hard manual tasks such as assembly line workers handling light objects, cooks, and hairdressers. | 1) Survival rate 2) Complications | “We conclude that this implant has acceptable long-term survival rate  and restores good hand function. We also report our methods to improve implant survival and to decrease the risk of component malpositioning, and failure rate.” |
| **Gómez-Garrido, 219** | ARPE TMC prosthesis | Total joint replacement | 137 | IV | 61.6 | 24 | 1) Patients with osteoarthritis of  the trapeziometacarpal joint 2) failure of conservative treatment in patients with TMC | 1) Revision rate  2) DASH | “The TMC joint prosthesis is an option for patients with TCM osteoarthritis, provides satisfactory outcomes and has a low failure rate” |
| **Semere et al, 2015** | Roseland prosthesis | Total joint replacement | 51 | IV | 71.3 | 150 | 1) All patients with CMC osteoarthritis who underwent CMC joint replacement using the Roseland HAC1 prosthesis in our department between 1996 and 2002 | 1) Radiographic assessment 2) Complications 3) ROM | “The long-term results with the Roseland1 HAC prosthesis are satisfactory in terms of pain relief and function. However, the high early complication rate is a major concern. We believe that the designs of CMC implant can still be improved, aiming at reducing the loads and increasing the stability of the proximal implant.” |
| **Zollinger et al, 2008** | Roseland prosthesis | Total joint replacement | 27 | IV | NS | 39 | 1) Patients with  complaints of osteoarthritis of the trapeziometacarpal or first carpometacarpal (CMC I) joint | 1)VAS 2) Satisfaction | “In all our patients Vitamin C 500 mg daily was started two days before surgery and continued for 50 days. There were no cases of CRPS under vitamin C prophylaxis. These results justify further investigation in a randomized clinical trial” |
| **Kollig et al, 2017** | Moje Acamo first carpometacarpal prosthesis | Total joint replacement | 28 | IV | 62 | 50 | 1) Patients with symptomatic  osteoarthritis of the thumb CMC joint who failed to respond to a minimum of 6 months of non-operative treatment including activity modification, oral analgesics or intra-articular steroid or hyaluronic acid  injections | 1) DASH score 2) Radiographic assessment | “In conclusion, the Moje Acamo CMC 1 prosthesis appears to be another implant with a high rate of failure. Based on our own findings, as well as on other published data and recommendations (Giddins,  2012), the use of this implant in its present configuration and coating should be suspended or limited to  ethically approved research studies.” |
| **Dehl et al, 2017** | Rubis II prosthesis | Total joint replacement | 95 | IV | 61 | 120 | 1)Patients with Rubis II prostheses were implanted | 1) DASH score 2) VAS score 3) Satisfaction 4) Revision rate 5) ROM | “This study confirms that the good clinical results of the Rubis II prosthesis are maintained in the medium and long term and represents a useful alternative to trapeziectomy for selected patients.” |
| **Thillemann et al, 2016** | Motec titanium carpometacarpal prosthesis | Total joint replacement | 40 | IV | 59 | 26 | 1) Patients with  uncemented thumb CMC joint prostheses. | 1) DASH score 2) NRS 3) Survival rate 4) Revision rate | “In conclusion we report unacceptably high revision rates. However, our findings suggest that when the prostheses need to be revised, implant removal and  trapeziectomy reveals an acceptable outcome. The release of metal particles does not seem to be associated with revision in this short-term study, but the long-term effects of local metal particles in CMC joint MoM prosthesis is unknown. Therefore, further long-term follow-up is warranted. We find it necessary to continue the development and research on CMC joint prosthesis, and advocate that the prostheses are only implanted in a carefully monitored setting with proper audit of the results.” |
| **Hansen et al, 2013** | Motec titanium carpometacarpal prosthesis, Motec PE cemented prosthesis, Elektra PE cemented prosthesis, Elektra bimetal prosthesis, Elektra chrome-cobalt prosthesis | Total joint replacement | 112 | III | 60.8 | 34.6 | 1) All patients who received TMC joint MOM and MOP total joint arthroplasties  from August 2004 to June 2011 for osteoarthritis. 2) All patients with the implants still in situ. | 1) DASH score 2) Revision rate | “We recommend that patients with trapeziometacarpal total joint replacement with metal-on-metal articulation is followed with DASH score and radiological examination every 3–5 years and serum chrome and cobalt should be analyzed in symptomatic cases to learn more about possible local complications leading to, or arising from, metal debris.” |
| **Hansen and stilling, 2013** | Elektra PE cemented prosthesis | Total joint replacement | 28 | I | 56 | 24 | 1) Patients with Eaton stage-2 or -3 osteoarthritis of the trapeziometacarpal  joint | 1) Revision rate  2) Grip strength 3) Pain 4) DASH | “Early implant fixation and clinical outcome were equally good with both cup designs. This is the first clinical RSA study on trapezium cups, and the method appears to be  clinically useful for detection of loose implants” |
| **Klahn et al, 2012** | Elektra chrome-cobalt uncemented prosthesis | Total joint replacement | 37 | IV | 56.5 | 48 | 1) Patients were treated with the second  generation of the Elektra prosthesis | 1)VAS score 2) Grip and pinch strength 3) Revision rate | “A third-generation Elektra has been designed, in which threading for cup fixation is avoided and a titanium cup is used. Time will show to what extent this will increase the durability of cup fixation. However, as a consequence of our experience with the poor durability of the Elektra prosthesis, our primary method of treating trapeziometacarpal osteoarthritis is once again  resection of trapezium and suspension arthroplasty.” |
| **Ulrich-Vinther et al, 2008** | Elektra chrome-cobalt uncemented prosthesis | Total joint replacement | 42 | III | 62 | 12 | 1) Patients with severe trapeziometacarpal joint osteoarthritis (Eaton-Littler stage 2.4 ± 0.1) | 1) Complications  2) ROM 3) Radiographic parameters | “This study demonstrates that patients with joint prostheses achieve faster convalescence with better patient comfort and improved strength and range of motion without any increased risk of complications than do patients treated with tendon interposition arthroplasty at 1-year follow up. However, a randomized clinical trial with long-term follow-up is required.” |
|  | Arthroplasty |  | 70 |  | 62 |  |  |  |  |
| **Regnard, 2006** | Elektra chrome-cobalt uncemented prosthesis | Total joint replacement | 100 | IV | 59 | 53 | 1) Patients with the Elektra trapeziometacarpal prosthesis is a cementless, unconstrained prosthesis made by  Fixano with titanium and chrome–cobalt steel | 1) Complication 2) Revision rate 3) Pain 4) Strength 5) Speed of recovery  6) Mobility | “The association of scaphotrapeziotrapezoidal osteoarthritis and the trapeziometacarpal arthritis is common but we think the latter joint alone may often be treated  by prosthetic replacement because the implantation is done with low tension, and this is sufficient to resolve the problem of both joints. We try to identify the importance of the scaphotrapeziotrapezoidal osteoarthritis before implantation and we explain to the patient  about the possibility of having to treat both joints. However, we feel that it is better to treat the scaphotrapeziotrapezoidal joint by a second procedure in the few cases in which pain recurs after some months.” |
| **Kirkeby et al, 2021** | Elektra | Total joint replacement | 89 | IV | 55 | 60 | 1) Who underwent a trapeziometacarpal total joint arthroplasty in the form of a ball-and-socket implant  type between 2003 and 2015 | 1) Revision rate 2) Survival rate | “In conclusion, our results, taking into account implant type, did not indicate large effects of higher occupational hand force requirements on revision rates of trapeziometacarpal total joint replacement arthroplasty.” |
|  | Motec |  | 47 |  |  |  |  |  |  |
|  | Moovis |  | 86 |  |  |  |  |  |  |
| **Froschauer et al,2019** | Elektra chrome-cobalt uncemented prosthesis | Total joint replacement | 32 | III | 54 | 159 | 1) Radiographic stage 3–4 thumb CMC OA and failed non-surgical treatment were indications for surgery. | 1) DASH 2) VAS 3) Strength 4) survival | “In conclusion, we cannot recommend Elektra prosthesis implantation for thumb CMC arthrosis because a significantly higher grip strength cannot outweigh such a high complication and revision rates due to aseptic cup loosening. Nevertheless, if  a prosthesis system could overcome the hurdle of aseptic cup loosening, total thumb CMC arthroplasty  could become a reasonable therapeutic option for thumb base surgery. Therefore, randomized studies with a comparable long-term follow-up period are needed to verify the sustainability of such a CMC prosthesis.” |
|  | Resection-suspension arthroplasty |  | 13 |  | 58 |  |  |  |  |
| **Bricout and Rezzouk, 2016** | MAÏA prosthesis | Total joint replacement | 139 | IV | 62.7 | 37.8 | 1) Patients with Maia prostheses were implanted | 1) DASH score 2) Complications 3) Revision rate 4) Radiological results | “The Maia1 trapeziometacarpal joint prosthesis provides  satisfactory outcomes and has a low failure rate. The main complications were prosthetic dislocation, trapezial loosening  and tendinopathy. It appears that the failures in our study were  mainly due to errors during the surgical implantation, as  opposed to inherent defects in the implant. Poor trapezial bone  quality discovered intraoperatively is a contraindication for  implantation of this non-cemented implant. The impact of any  MCP deformity must not be underestimated, and it must be  dressed aggressively. The condition of overlying and underlying joints must also be considered when selecting a treatment: MCP stiffness or fusion are presumed loosening risks because of excessive loads; radiological damage of the STT joint is a source of residual pain.” |
| **Toffoli and Teissier, 2017** | MAÏA prosthesis | Total joint replacement | 80 | IV | 68 | 76 | 1) Patients who underwent MAÏA TMC prosthesis implantation | 1) Kapandji score 2) Strength 3) Pain 4) DASH | “MAÏA TMC total joint arthroplasty may be a reliable treatment option for TMC joint osteoarthritis, with very good results for pain relief, strength, mobility, and restoration of  the thumb length, providing correction of most thumb z-deformities.” |
| **Caekebeke et al, 2017** | MAÏA prosthesis | Total joint replacement | 35 | IV | 57 | 56 | 1) Patients treated with a trapeziometacarpal prosthesis at us institution from May 2011 to May 2012. | 1) Survival rate 2) DASH 3) Kapandji score 4) VAS 5) Satisfaction | “We agree with previous reports that meticulous surgical technique is important in total trapeziometacarpal joint arthroplasty. Correct position of the  cup is the most difficult to judge. Optimal orientation of the cup in the trapezium within a non-constrained prosthesis could contribute to good results in the  long term. We advise the use of intra-operative fluoroscopy to optimize the cup position.” |
| **Andrzejewski et al, 2019** | MAÏA prosthesis | Total joint replacement | 93 | IV | 59.3 | 60 | 1) Patients with MAÏA trapeziometacarpal  joint arthroplasty | 1) Complications 2) DASH 3) Survival rate 4) Strength | “The medium-term results with the Maia prosthesis are satisfactory. However, the high complication and revision rates are a major concern and care must be taken in the future to avoid early dislocations and periprosthetic ossifications” |
| **Lemoine et al, 2009** | GUEPAR prosthesis (second generation) | Total joint replacement | 57 | IV | 55 | 61 | 1) Patients with GUEPAR II prostheses were implanted to treat advanced and severely incapacitating first CMC osteoarthritis. | 1) Revision rate 2) Pain 3) Force 4) ROM 5) Patient satisfaction | “In our series, GUEPAR II total joint arthroplasty of the thumb CMC joint has proven to be efficacious, improving motion, strength, and achieving a high degree of pain relief. Successful outcome appears in our experience contingent upon strict compliance with numerous  surgical technique details. Current research focuses on improving bipolar fixation by developing  press-fit cementless implants.” |
| **Pendse et al, 2009** | Avanta SRTM TMC prosthesis | Total joint replacement | 50 | IV | 64.5 | 36 | 1) Patients undergoing  trapeziometacarpal joint arthroplasty using the SR TMC prosthesis over a 5-year period | 1) Survival 2) Revision rate 3) Complications 4) Strength 5) VAS 6) DASH 7) Kapandji score 8) Radiological results | “We can therefore reliably comment that apart from the two failures (one revision and one trapezial loosening) the remaining patients were satisfied with the procedure” |
| **Badia, 2006** | Braun-Cutter TMC joint prosthesis | Total joint replacement | 25 | IV | 71 | 59 | 1) Patients who have the diagnosis of thumb basal joint arthritis who did not improve after conservative treatment underwent arthroscopy for further evaluation of the joint status and surgical treatment during the past 10 years. | 1) Radiographic assessment 2) Clinical assessment | “Arthroscopic assessment of the CMC joint allows direct visualization of all components of  the joint, including synovium, articular surfaces, ligaments, and the joint capsule. It also allows for  the extent of joint pathology to be evaluated and staged with intraoperative management decisions made based on this information. The author recommends this arthroscopic staging to ensure better judgment of this condition in order to provide the most adequate treatment option to patients who have this disabling condition.” |
| **Hannula and Nahigian, 1999** | Cementless trapeziometacarpal prosthesis | Total joint replacement | 36 | IV | 58 | 47 | 1) Patients with a cementless ball and socket trapeziometacarpal arthroplasty was used for the treatment of  Eaton and Littler stage II and III trapeziometacarpal osteoarthritis. | 1) Revision rate 2) Radiographic assessment  3) Pain relief 4) Strength | “The cementless arthroplasty failures can be effectively salvaged by converting them to ligament reconstruction tendon interposition arthroplasty” |
| **Cooney et al, 1987** | Mayo Implant | Total joint replacement | 57 | IV | 62 | 55.2 | 1) Severe degenerative arthritis that was restricted to the TMC joint | 1) ROM 2) Strength | “The authors continued selected use of cemented trapezial prostheses in the hand but cannot recommend unrestricted release and use of these implants at this time” |
| **Ferrari and Steffee, 1986** | Steffee prosthesis | Total joint replacement | 38 | IV | 61 | 51 | 1) Patients received the cemented total joint replacement for treatment of painful trapeziometacarpal arthritis that was refractory to nonoperative management. | 1) Radiographic assessment 2) ROM 3) Strength | “We concluded that  trapeziometacarpal total joint replacement can provide  good relief of pain and restore function of the thumb to  patients with severe trapeziometacarpal arthritis, although further study is necessary to assess the long-term results of the procedure” |
| **Gonzalez-Espino et al, 2021** | TOUCH® Prosthesis | Total joint replacement | 92 | III | 62.24 | 16 | 1)Patients underwent CMC joint replacement by Touch1 prosthesis,  between July 2018 and March 2020. All showed Dell stage 3–4 on preoperative X-ray | 1) DASH 2) Satisfaction 3) NRS 4) VAS 5) Complications | “It now seems that the TOUCH1 dual mobility prosthesis is a safe  and reliable implant that gives high satisfaction with functional  scores comparable to those of the general population. It also allows  fast return to work and leisure activities. Dual mobility ensures  better stability than previous designs. The present study needs  further follow-up to assess the long-term complications such as  implant loosening. Considering the high rate of De Quervain’s  tenosynovitis, we suggest opening the first extensor compartment  and releasing the abductor pollicis longus and extensor pollicis  brevis to prevent the occurrence of De Quervain’s tenosynovitis, as it  is a debilitating disease that jeopardizes the success of TMC surgery” |
| **Lussiez et al, 2021** | TOUCH® Prosthesis | Total joint replacement | 107 | IV | 64.5 | 40 | 1) Patients with  TMC arthritis who were operated by one of the three surgeons, 2) Who received the Touch prosthesis, and  3) Who agreed to a clinical and radiological follow-up at 1, 3, 5, 10, and 15 years. | 1) DASH 2) Kapandji score 3) VAS 4) Strength | “In conclusion, our preliminary cohort of 107 cases using a dual mobility TMC prosthesis with more than 3 years of follow-up have shown promising results, with no incidence of early prosthetic dislocations, rapid recovery of strength and range of motion. A high satisfaction rate was achieved in 95% in our cohort, with rapid return to work for 30 of 34 patients. Survival of prostheses in our cohort are similar to published series of metalon-polyethylene prostheses with long-term follow-up. Minor radiological modifications have been noted around the cup and stem, without clinical consequences after 3 years. These modifications justify close monitoring of the prosthesis over the longer term.” |
| **Froschauer et al, 2021** | TOUCH® Prosthesis | Total joint replacement | 37 | IV | 57.7 | 12 | 1) Patients suffering from symptomatic thumb CMC OA with radiographic Eaton–Littler stage III and failed non-surgical treatment | 1) DASH 2) Strength 3) Complications 4) VAS 5) ROM | “Despite the occurrence of some  complications, we recommend implantation of this prosthesis type due to favorable clinical and  radiological performance.” |
| **Dremstrup et al, 2020** | Moovis cup and the Elektra stem | Total joint replacement | 168 | II | 59 | 24 | 1) Patients who were  scheduled for surgery with the cementless press-fit Moovis cup and the Elektra stem. Patients were enrolled from February 2013 to February 2017 | 1) Strength 2) DASH 3) Pain 4) Survival rate 5) Complications | “We conclude that early outcomes of this new-generation implant is favorable, but its surgery is challenging and associated with important intraoperative and early postoperative complications related to the learning curve. Attentive reaming of the trapezium and careful cup impaction are crucial steps to avoid trapezial fracture and early cup failure.” |
| **Dreant et al, 2018** | Moovis TMC prosthesis | Total joint replacement | 25 | III | 63.4 | 27.5 | 1) Patients with  Eaton and Littler stage III CMC osteoarthritis, who benefited from a CMC joint replacement using the MOOVIS  prosthesis between March 2014 and November 2016, | 1) VAS 2) DASH 3) ROM 4) Strength 6) Pain | “Total joint arthroplasty with a dual mobility prosthesis appears to be a satisfactory solution in our series. The absence of prosthesis instability encourages us to recommend this technique for the treatment of advanced trapeziometacarpal osteoarthritis for people having an activity without too many manual constraints.” |
| **Tchurukdichian et al, 2021** | Moovis TMC prosthesis | Total joint replacement | 179 | II | 66 | 48.2 | 1) Patients who  underwent double-mobility TMC prosthesis implantation | 1) VAS 2) DASH 3) Survival 4) ROM 5) Radiographic assessment 6) Strength 7) Kapandji scale | “After a minimal of 3 years of follow-up, prosthesis with double mobility seemed to bring better stability in implant for thumb prosthetic replacement.” |
| **Martins et al, 2020** | Moovis TMC prosthesis | Total joint replacement | 46 | IV | 68 | 60 | 1) Patients with Moovis prostheses implanted between April 2012 and January 2017 by the same surgeon | 1) DASH 2) Pain 3) Strength 4) Radiographic results | “We conclude the Moovis is a reliable and effective implant at short- to mid-term follow-up” |
| **Mosegaard et al, 2020** | Elektra Bimetal cementless cup | Total joint replacement | 62 | II | 58.8 | 12 | 1) All patients having a total joint replacement of the TMC joint due to osteoarthritis. | 1) VAS 2) DASH 3) Strength | “However, we were unable to detect one isolated preoperative predictor as indicator of successful result after operative treatment of TMC osteoarthritis, and as so it was not possible to establish a clinical valid tool for patient selection before surgery” |
|  | Moovis press-fit dual-mobility cementless cup |  | 142 |  | 58.5 |  |  |  |  |
|  | Elektra cemented polyethylene cup |  | 10 |  | 57.9 |  |  |  |  |
|  | Motec cemented polyethylene |  | 41 |  | 60.5 |  |  |  |  |
|  | Motec cementless titanium cup |  | 20 |  | 60 |  |  |  |  |
|  | Elektra cementless cup. |  | 12 |  | 60.4 |  |  |  |  |
| **Stillwater, 2017** | Pyrohemisphere TMC prosthesis | Hemiarthroplasty | 26 | IV | 58 | 13 | 1) Patients with first CMC joint Pyrohemisphere™ implant arthroplasty | 1) Radiographic assessment | “Although radiography may be useful in the correct clinical context, it should not be utilized as the sole predictor of adverse clinical outcomes following carpometacarpal arthroplasty.” |
| **Martinez de Aragon et al, 2009** | Pyrohemisphere TMC prosthesis | Hemiarthroplasty | 49 | IV | 59 | 20 | 1) Patients were treated with use of a pyrolytic carbon hemiarthroplasty procedure. | 1) Survival rate  2) Grip strength 3) Pinch strength 4) Satisfaction | “Pyrolytic carbon thumb arthroplasty may prove to be an acceptable option for the treatment of TM, although a high complication rate was observed in this early cohort, with many cases of subluxation attributed to the creation of a too shallow trapezial cup. Further comparative studies are warranted.” |
| **Pritchett and Habryl, 2012** | BioPro Modular thumb prosthesis | Hemiarthroplasty | 124 | IV | 63 | 72.1 | 1) Selected patients with Eaton-Littler Stage II or III  osteoarthritis of the trapeziometacarpal joint who underwent thumb basal joint hemiarthroplasty using the BioPro1  Modular Thumb prosthesis between 2001 and 2008. | 1) Pain 2) Strength 3) ROM 4) Tip pinch | “Our results are superior to those of other implants and support continued use of this implant. Studies with longer follow-up are required to confirm these results” |
| **Naidu et al, 2006** | Swanson titanium convex condylar prosthesis | Hemiarthroplasty | 47 | IV | 55 | 24 | 1) Convex condylar titanium basal joint implant arthroplasties were performed | 1) Grip strength 2) Key pinch 3) DASH | “Anecdotal quotations show success rates for titanium implant arthroplasty for basal joint arthritis to be as high as 97%. Our results are quite to the contrary in that high  failure rates were common early in the follow-up period. Our FEA results are confirmed by the clinical study. Titanium implant arthroplasty may have a role in low-demand patients with good bone stock, however, we have stopped offering titanium hemiarthroplasty to patients at our institution.” |
| **Conolly and Lanzetta, 1993** | Silicone Metatarsophalangeal implant | Hemiarthroplasty | NS | III | 54.5 | 51.6 | 1) Patients had isolated trapezio-metacarpal joint arthritis. | 1) Satisfaction 2) Revision rate | “In conclusion, recommendations for the treatment of arthritis of the carpometacarpal joint of the thumb are as follows: for trapezio-metacarpal osteoarthritis, arthrodesis, or silicone hemiarthroplasty; for pan-trapezia1 osteoarthritis, silicone arthroplasty or soft tissue arthroplasty; and for scapho-metacarpal osteoarthritis, a silicone metatarso-phalangeal implant.” |
| **Jennings and Livingstone, 1990** | Convex condylar silicone implant | Hemiarthroplasty | 25 | III | 63.3 | 54 | 1) Convex condylar arthroplasties were done, all with HP silicone elastomer. | 1) Radiographic findings 2) ROM 3) Revision rate 4) Grip strength 5) Pinch strength 6) Pain | “It should be emphasized that all the failures in this series and in the other series of convex condylar implants have been in the osteoarthritic thumb and not in the rheumatoid thumb. This would imply that load is a significant factor with regard to failure. In spite of many well-functioning thumbs, radiographic findings  of metacarpal bone resorption in 84% of osteoarthritic thumbs suggest that more failures may occur in the future. We will continue to closely follow these patients clinically and radiographically. Patients with these changes seen on x-ray films should be told of the probable need for revision. We would agree with Pelligrini and Burton that "the Swanson condylar hemitrapezium replacement (be reserved) for the low demand rheumatoid thumb.:" Fusion to the remaining trapezium or tendon interposition arthroplasty have provided successful options for revision.” |
| **Howard et al, 1985** | Convex condylar silicone implant | Hemiarthroplasty | 30 | IV | 62.3 | 15.5 | 1) Patients with disabling arthritis. | 1) Pain  2) Complications | “The authors present 40 cases of the Silastic condylar arthroplasty for arthritis of the first carpometacarpal joint with preservation of trapezium. Changes in the surgical technique have resulted in more stability of the implant.” |
| **Swanson et al, 1981** | Convex condylar silicone implant | Hemiarthroplasty | 121 | IV | 54 | 28 | 1) Patients with  thumbs treated from 1965 to 1976 by flexible implant  resection arthroplasty of the trapezium | 1) ROM 2) Strength 3) Radiographic review 4) Complications | “Patients have been able to return to normal activities, including physically stressful work and  sports. If certain technical considerations in the treatment program are carefully adhered to, predictably good and reproducible results will be easily obtained.” |
| **Aita et al, 2016** | Ascension implant | Hemiarthroplasty | 45 | II | 63.2 | 42.1 | 1) Patients with a diagnosis of idiopathic rhizarthrosis who underwent resection arthroplasty and interposition of an uncemented Ascension® implant, made of pyrocarbon. | 1) ROM 2) VAS 3) DASH 4) Complications | “This method is effective for treating rhizarthrosis, according to the measurements made on the clinical and functional results, even after taking the complication rate  into consideration. Moreover, it provides an improvement of quality of life for these patients.” |
| **Russo et al, 2016** | Pyrocardan implant | Interposition With Partial Trapezial Resection | 36 | IV | 58.5 | 31.5 | 1) Patients with TM arthritis were treated using Pyrocardan implant arthroplasty. | 1) DASH 2) VAS | “TM arthroplasty with Pyrocardan implantation is a suitable option in treating Eaton-Littler grade II or III TM osteoarthritis.” |
| **Gerace et al, 2020** | Pyrocardan implant | Interposition With Partial Trapezial Resection | 96 | IV | 59 | 67 | 1) All consecutive patients with painful  TMC OA treated from March 2009 to October 2014, in a single  center and after failure of a minimum of 6 months of conservative  treatment. Radiographically, patients had Eaton-Littler grade  I, II or early grade III OA. | 1) DASH 2) PRWHE 3) Strength 4) ROM | “In summary, with a long-term follow-up, Pyrocardan arthroplasty for TMC OA provides pain relief, good strength, and good  thumb function recovery with a high survival rate. Clinical and  radiological results do not seem to deteriorate over time. This  makes it a reliable alternative to trapeziectomy or total prosthesis  especially for young active patients. Subluxation of the M1 base is  not a contraindication for this minimally invasive arthroplasty.  Longer follow-up and prospective comparative studies are needed to confirm all these findings.” |
| **Logan et al,2020** | Pyrocardan implant | Interposition With Partial Trapezial Resection | 37 | III | 58 | 24 | 1) Pyrocardan trapeziometacarpal implantation for Eaton and Littler grade 1,  2, and 3 arthritis with mild-to-moderate joint subluxation | 1) VAS 2) Satisfaction 3) DASH 4) PRWHE 5) Strength 6) Kapandji score | “Pyrocardan interposition arthroplasty appears to be a safe, effective treatment for trapeziometacarpal arthritis. Patient-reported clinical outcomes were at least equivalent to LRTI and are comparable to Bellemère’s original series. Grip and pinch strength appear to be better than LRTI” |
| **Odella et al, 2014** | Pyrocardan Implant, PyroDisk | Interposition With Partial Trapezial Resection | 59 | IV | 62 | 42 | 1) Patients surgically treated with TMC interposition arthroplasty with pyrolytic implants from 2007 to 2014 | 1) DASH 2) VAS | “Prosthetic replacement of the TMC joint was found to be a good solution for low demand patients. However, the PyroDisk could be a good solution in selected patients (Eaton stage I-III, non-subluxated joint): it provides good pain relief, good range of motion, good pinch, and grasp strength, and stable results at more than three-years of follow-up.” |
| **Mariconda et al, 2014** | PyroDisk | Interposition With Partial Trapezial Resection | 25 | IV | 63 | 34 | 1) PyroDisk implants in patients for advanced osteoarthritis of the TMC joint (Eaton stage II or III). | 1) DASH 2) Pain 3) VAS 4) Pinch 5) Kapandji test | “Partial trapeziectomy with pyrocarbon arthroplasty may prove to be a successful option for the treatment of trapeziometacarpal joint osteoarthritis. Further long-term comparative studies are warranted.” |
|  | Control |  | 27 |  |  |  |  |  |  |
| **Oh et al, 2019** | PyroDisk | Interposition With Partial  Trapezial Resection | 20 | III | 63.3 | 35.2 | 1) All patients with  Eaton-Littler stage II or III TMC arthritis  2) Who underwent  trapezium excision with LRTI or PyroDisk interpositional  arthroplasty from March 2009 to August 2014 3) Had 2 or more years of follow-up. | 1) DASH 2) VAS 3) Kapandji score 4) Strength | “All subjective and objective outcomes were similar following LRTI and pyrolytic interpositional arthroplasty in patients with TMC arthritis, except pinch strength, which was more improved following pyrolytic interpositional arthroplasty. Longer follow-up is required to test adverse effects of high rates of periprosthetic lucency and prosthetic subluxation on clinical outcomes after PyroDisk interpositional arthroplasty” |
|  | LTRI |  | 19 |  | 58.9 | 40.6 |  |  |  |
| **Smeraglia et al, 2020** | PyroDisk | Interposition With Partial  Trapezial Resection | 46 | IV | NS | 113 | 1) All patients who received a PyroDisk implant | 1) DASH 2) VAS 2) Key pinch | “In conclusion, the long-term follow-up reported in  this article indicates that this implant is reliable. Review of literature revealed that the functional outcomes after implant surgery are not superior to more common techniques, such as trapeziectomy with or without ligamentoplasty. Therefore, this surgery may not have added benefits over simpler surgical treatments. The implant surgery has a higher cost. The clinical values of this procedure may be questioned.  When we compare the results of our study to the results of previous studies, we find similar objective  and subjective outcomes.” |
| **Diacom et al, 2011** | Polylactic acid implant | Interposition With Partial Trapezial Resection | 25 | IV | 64.5 | 14 | 1) Patients, presenting with osteoarthritis of the trapeziometacarpal joint that had been  medically treated for 18.5 months on average. 2) All patients were operated using 1-ulnar (U) and 1-radial (R) portals. After joint debridement, a polylactic acid implant was inserted under arthroscopic control. | 1) Pain 2) Grip strength 3) Pinch Strength 4) ROM | “Our technique is simple, rapid, cost-effective, and does not necessitate trapeziectomy, even partial. It has the same indications as other non-radical interventions. The follow-up duration of our study was too short for long-term evaluation, but short-term outcome appeared superior to that in other published series. The regional inflammatory reactions that occurred in our series were transient and probably related to implant resorption. Our promising results suggest extending the indication of arthroscopic interposition to more advanced stages of proximal joint osteoarthritis.” |
| **Blount et al, 2013** | Artelon Spacer | Interposition With Partial Trapezial Resection | 32 | III | NS | 30 | 1) Patients with CMC arthritis having either placement of  Artelon spacer or trapeziectomy and LRTI. | 1)VAS 2) MHQ | “In our practice, use of the Artelon joint spacer resulted in an explantation rate of 37%.  Due to these findings, we have abandoned its use for treatment of basilar thumb osteoarthritis. In  contrast to previous studies, pain and satisfaction are worse in patients with intact Artelon spacers  than those who had received LRTI.” |
|  | Control with LRTI |  | 10 |  |  |  |  |  |  |
| **Clarke et al, 2011** | Artelon Spacer | Interposition With Partial Trapezial Resection | 29 | IV | 51 | 8 | 1) Patients who underwent Artelon  CMC arthroplasty | 1) Complications  2) Revision rate | “Our study found a significant short-term complication rate following Artelon spacer arthroplasty of the CMC joint. This is higher than previously described.  We could not identify any factors that were significantly associated with the complications. It is possible that the inherent instability of the joint or the material of the spacer is involved in implant failure. Further study is necessary to better define the indications for use and specific techniques  for the use of the implant.” |
| **Bell et al, 2011** | Artelon Spacer | Interposition With Partial Trapezial Resection | 46 | IV | 57.8 | 48 | 1) Patients with Eaton stage I, II, or III carpometacarpal (CMC) joint arthritis and disabling pain that had failed conservative treatment | 1) DASH  2)VAS  3) Strength 4) Satisfaction | “At a follow-up period of 4 years, patients had measured improvement as well as positive self-reported functional improvements following Artelon® implant insertion for the treatment of arthritis of the thumb CMCJ” |
| **Mattila et al, 2021** | Artelon Spacer | Interposition With Partial Trapezial Resection | 22 | IV | 54 | NS | 1) All implant procedures performed for primary TMC osteoarthritis (Eaton-Glickel stages 2-3) with no radiological evidence of osteoarthritis in the scaphotrapeziotrapezoidal joint, at a single institution (Helsinki University Hospital,  department of hand surgery) during the years 2003 to 2013. | 1) Revision rate | “Interposition implant arthroplasty may yield high revision rates. The results after revision surgery may be worse than previously described, and there may also be a tendency for worse results than those of primary arthroplasty. Interposition implant arthroplasty should always be thoroughly contemplated.” |
| **Nilsson et al, 2010** | Artelon Spacer | Interposition With Partial Trapezial Resection | 72 | I | 60 | 12 | 1) Patients with painful and radiographically verified OA (Eaton stage 1–3) in the CMC joint. | 1)DASH 2) VAS 3) Strength | “The Artelon CMC spacer did not show superior  results compared to tendon interposition arthroplasty. Proper  use of preoperative antibiotics and a thorough patient selection  appear to be important for the results.” |
|  | Control group with tendon arthroplasty |  | 37 |  |  |  |  |  |  |
| **Bengezi and Vo, 2014** | Pyrocarbon spherical implant | Interposition With Partial Trapezial Resection | 23 | IV | 56 | 18.2 | 1) Clinical findings of a positive grind test 2) Persistent tenderness in the first CMC region 3) Dorsal-radial prominence (subluxation) of the base of the first metacarpal bone 4) Decreased range of motion and pinch  Strength.  5) Radiographic findings included Eaton-Littler stage II or III OA of the first CMC. | 1) Patient satisfaction 2) Radiographic assessment  3) The DASH score | “The biocompatibility of pyrocarbon and the wide range of motion of the first CMC make the spherical-shaped pyrocarbon implant the implant of choice when performing arthroplasty of the first CMC in patients with Eaton-Littler stage II and III OA. Early outcomes show promising results and support the use of this implant for arthroplasty. However, longer-term follow-up will be needed to confirm these results” |
| **van Laarhoven et al, 2020** | Pyrocarbon disc | Interposition With Partial  Trapezial Resection | 137 | IV | 58 | 84 | 1) Patients who performed pyrocarbon disc interposition arthroplasties after distal hemitrapeziectomy for CMC thumb joint arthritis. | 1) ROM 2) PRWHE 3) DASH 4) MHQ 5) Satisfaction 6) Radiographic assessment 7) Revision rate 8) Complications | “Our study suggests that pyrocarbon disc interposition arthroplasty is a reliable and  feasible treatment for carpometacarpal thumb joint arthritis at medium-term follow-up. It was associated with a high level of patient satisfaction; it maintained thumb height and the implant  survived in 91% of patients. Strength and range of motion were comparable to the contralateral  hand after a minimum follow-up of 5 years.” |
| **Adams et al, 2009** | Orthosphere | Interposition With Partial Trapezial Resection | 49 | IV | 59 | 36 | 1) Patients with ceramic, spherical implants for the treatment of trapezialmetacarpal osteoarthritis | 1) Pain relief  2) Thumb strength 3) Patient satisfaction 4) Radiographic outcomes 5) Complications | “Although most patients were satisfied with their function and pain relief, adverse radiographic findings were nearly universal. Implant subsidence, especially into the trapezium, was often severe, with some resulting in a trapezium fracture. Based on the radiographic outcome at this medium-term follow-up, we no longer use this implant.” |
| **Ashworth et al, 1977** | Ashworth implant | Interposition With Partial Trapezial Resection | 42 | IV | 55 | 31 | 1) Patients with interposition arthroplasty procedures at the carpometacarpal joint of the thumb | 1) Grip strength 2) Pain | “The advantages of interposition arthroplasty of the carpometacarpal joint of the thumb has been presented. The procedure has been performed 49 times. Follow-up has ranged from 6 to 75 months, with a mean follow-up time of 31 months. Forty were rated excellent, six good, one fair, and one poor. The poor result was revised with final rating of good. The method has the following advantages: (1) preservation of anatomical relationship by minimal resection; (2) the dorsoradial facet of the trapezium is replaced by the surface of the disc; (3) the implant is less subject to disruption or dislocation than some of the other types of trapezial prostheses; (4) the procedure is such that should a complication occur and additional operations be required, all other alternative procedures still are possible.” |
| **Engel et al, 1982** | Kessler implant | Interposition With Partial Trapezial Resection | 23 | IV | 59.3 | 24 | 1) Patients who underwent operations for insertion of Kessler’s implant during 1973-1978, | 1) Pain 2) Power of pinch 3) Power of grasp 4) ROM | “It is concluded that the replacement of the metacarpal articular surface of the first carpometacarpal joint with a silicone implant has given  in our experience a high rate of pain relief and improvement in ability to carry out daily activities. The method is relatively simple and reliable and certainly deserves further application so that more experience in its use can be gained.” |
| **Kessler et al, 1984** | Kessler implant | Interposition With Partial Trapezial Resection | 40 | IV | 40 | 24.7 | 1) Patients after trapeziectomy for either osteoarthritis, rheumatoid arthritis, or trauma with stemless Proplast*-covered silicone rubber trapezium implants | 1) Revision  2) ROM | “In conclusion, a trapezium implant that combines the unique features of Proplast and silicone rubber was designed so that it could become stabilized without the need of a stem. Theoretically, the stemless feature allows the metacarpal a satisfactory ROM without projecting the generated force to the capsule that covers the implant. In practice, this has clinically worked well in a series of 42 patients without any wound complications, significant improvement in comfort and pinch strength, and the maintenance of good active ROM.” |
| **Mattila et al, 2017** | RegJointTM  interposition | Interposition With Partial Trapezial Resection | 23 | IV | NS | 40 | 1) Patients with radiologically verified isolated TMC OA stage 2 or 3 were operated on by partial trapeziectomy and  implantation of the RegJointTM joint scaffold, which  is made of PLDLA copolymer with an L/D isomer ratio of 96/4 | 1) DASH 2) VAS 3) PEM 4) Kapandji score 5) Strength | “Owing to the high incidence of adverse tissue reactions, the use of the implant has been discontinued in the treatment of trapeziometacarpal osteoarthritis.” |
| **Sander et al, 2020** | RegJointTM  interposition | Interposition With total trapeziectomy | 21 | III | 60.8 | 27.7 | 1) Thumb CMC joint osteoarthritis,  2) Severe pain limiting the patient activities of daily living  3) Eaton and Littler grade II-IV on preoperative X-rays 4) APL suspension interposition arthroplasty as chosen treatment, with the indication for surgery determined  primarily by clinical findings and not radiological grade | 1) ROM  2) Strength 3) DASH | “The modified APL suspension interposition arthroplasty was an efficient and simplified option for the treatment of thumb CMC joint osteoarthritis, with results comparable or better than other published procedures. The APL suspension technique was easy to perform avoiding difficult bone tunneling and incision of the FCR tendon. The RegJoint™ interposition as spacer prevented impingement of the first metacarpal base on the second metacarpal base or the trapezoid bone.” |
| **Mattila et al, 2019** | RegJointTM  interposition | Interposition With total trapeziectomy | 34 | IV | 62 | 33 | 1) Patients operated by total trapeziectomy and  Interposition of the RegJoint. | 1) DASH 2) VAS 3) Strength 4) Kapandji score 5) Strength | “We conclude that the use of RegJointTM should be reconsidered since the implant probably offers no benefit in the treatment of trapeziometacarpal osteoarthritis.” |
| **Kennedy et al,2019** | RegJointTM  interposition | Interposition With Partial Trapezial Resection | 22 | IV | 69.5 | 36.5 | 1) Patients were retrospectively identified from theatre log books. From January 2013 to July 2016, 25 RegJointTM procedures for CMCJ OA and 1 procedure for thumb MCPJ OA were performed in 22 patients. | 1) PEM 2) DASH 3) Strength 4) VAS | “A guiding principle in medicine is ‘‘primum non nocere’’ or first,  do no harm. We did not seek to investigate superiority of the  RegJointTM over other techniques for CMCJ arthritis but to demonstrate that this implant is safe and biologically inert. Our  results dispute the contention that the RegJointTM causes adverse  tissue reactions or significant bone osteolysis. Our study also  demonstrates that the implant causes no significant change in  hand function. We therefore still use the RegJointTM as a useful  adjunct in the management of a select cohort of patients with  challenging CMCJ arthritis.” |
| **Dietrich et al, 2021** | RegJointTM  interposition | Interposition With Partial Trapezial Resection | 22 | III | 58.17 | 44.76 | 1) Patients with symptomatic osteoarthritis resistant to conservative treatment underwent surgery in our department. | 1) DASH 2) VAS 3) PEM 4) Strength 5) Kapandji score 6) ROM | “We conclude that RegJointTM spacers do not produce more complications than suspension alone but provide no added benefit.” |
|  | Suspension alone |  | 12 |  | 58.79 | 95 |  |  |  |
| **Agout et al, 2016** | Pi2 | Interposition Complete Trapezial Replacement | 39 | IV | 63 | 125.49 | 1) Total trapeziectomies with Pi2 free pyrocarbon interposition implants | 1) ROM  2) Strength 3) Kapandji test | “Results at more than 10 years using the Pi2 free interposition  implant confirmed that this is a valid solution in the treatment of  advanced carpometacarpal osteoarthritis. This procedure allows early functional recovery that is maintained over the long term. A very precise surgical technique, notably in the ligament reconstruction phase, is indispensable to prevent dislocation and extend the current positive clinical results.” |
| **Szalay et al, 2013,** | Pi2 | Interposition Complete Trapezial Replacement | 60 | IV | 58.5 | 23.6 | 1) Patients with clinical complaints and a radiological stage 3 or 4 on Eaton’s scale | 1) Clinical outcome 2) Radiological assessment | “Considering the high rate of spacer dislocations, which does not appear to be related with the operating technique, added to the high cost of the implant, and the possibility to achieve comparable or even superior results with trapeziectomy or suspension  arthroplasty, we eventually discontinued using this implant.” |
| **Colegate-Stone et al, 2011** | Pi2 | Interposition Complete Trapezial Replacement | 24 | III | 62 | 12 | 1) All patients  undergoing surgery for osteoarthrosis of the trapeziometacarpal  joint | 1) Pain  2) Grip strength 3) DASH 4) VAS | “We suggest that pyrocarbon interposition does not significantly improve postoperative function, requires a longer operation with a high postoperative risk of pyrocarbon displacement and need for revision surgery” |
|  | Without implant | Trapeziectomy | 14 |  | 63.5 |  |  |  |  |
| **Van Aaken et al, 2016** | Pi2 | Interposition Complete Trapezial Replacement | 41 | IV | 60 | 29 | 1) Patients treated  for trapeziometacarpal osteoarthritis | 1) Revision rates  2) Clinical outcomes | “We have abandoned the use of the PI2® spacer. We recommend the establishment of a registry for evaluation of future implants.” |
| **Avisar et al, 2015** | Tie-In trapezial implant | Interposition Complete Trapezial Replacement | 22 | IV | 66 | 18 | 1) All patients who underwent trapeziectomy and tendon tie-in trapezium  implant arthroplasty stabilized with a Weilby flexor carpi radialis tendon sling for osteoarthritis of  the TMC joint | 1) Pain 2) Complications | “Good short-term to mid-term results and stability of TMC arthroplasty implant can be  achieved with tie-in trapezium implant stabilized with a Weilby flexor carpi radialis tendon sling” |
| **Lallemand et al, 2019** | Tie-In trapezial implant | Interposition Complete Trapezial Replacement | 21 | IV | 57 | 18 | 1) Patients with Tie in procedure were performed after failure of primary TMC surgery | 1) Survival | “Survival of the Tie-in silicone implant in TMC revision surgery is poor; nearly half of implants failed at 2 years. The rate of silicone synovitis is also important because future revision might be more complex owing to bone loss.” |
| **Spaans et al, 2014** | Polyethylene mesh implant | Interposition Complete Trapezial Replacement | 66 | IV | NS | 18 | 1)Adults with stage IV  trapeziometacarpal osteoarthritis had a trapeziectomy with interposition of a spacer made of  polyethylene terephthalate mesh | 1) Revision rate 2) Complications | “In the light of these results and the available literature, we recommend not using this material for interposition in the treatment of osteoarthritis of the trapeziometacarpal joint” |
| **Jewell et al, 2011** | Swanson trapezium implant | Interposition Complete Trapezial Replacement | 63 | IV | 66 | 46 | 1) All patients who received a silicone replacement of their trapezium | 1) Patient’ satisfaction 2) Pain 3) Key and power grip strength 4) Range of motion  5) Complications | “Silicone trapezium replacement is an effective operation for arthritis of the first carpometacarpal joint” |
| **Taylor et al, 2005** | Swanson trapezium implant | Interposition Complete Trapezial Replacement | 22 | III | 66 | 42 | 1) Patients who underwent surgery for the treatment of  osteoarthritis of the trapeziometacarpal joint | 1) Patients’ satisfaction  2) ROM 3) Strength 4) Pain | “We recommend excision of the trapezium with or without soft tissue reconstruction as the operation of choice in middle-aged to elderly patients. This recommendation is made purely  on the basis that this group had the lowest complication rates.” |
|  | Excision with or without sling ligament reconstruction |  | 25 |  | 63 | 33 |  |  |  |
|  | Fusion of CMJ |  | 36 |  | 64 | 39 |  |  |  |
| **MacDermid et al, 2003** | Swanson trapezium implant | Interposition Complete Trapezial Replacement | 25 | IV | 64 | 78 | 1) Patients with advanced osteoarthritis who underwent silicone rubber trapezial arthroplasty. | 1) Patients’ satisfaction  2) ROM 3) Strength 4) Pain | “Although clinical, functional, and radiographic results were poor, they  did not predict either satisfaction or pain improvement reported by patients, illustrating the need for a  comprehensive standardized outcome evaluation to make informed decisions on the value of surgical intervention for osteoarthritis of the trapeziometacarpal joint.” |
| **Bezwada et al, 2002** | Swanson trapezium implant | Interposition Complete Trapezial Replacement | 58 | IV | NS | 196 | 1) TMC joint silicone implants were placed in patients for severe TMC joint arthritis (Eaton stages 3 and 4) | 1) Radiographic assessments 2) Strength 3) Complications | “Trapeziometacarpal joint silicone arthroplasty seems to provide  good, long-term patient satisfaction and improved function with a low complication rate” |
| **van Cappelle et al, 2001** | Swanson trapezium implant | Interposition Complete Trapezial Replacement | 45 | IV | 61 | 165.6 | 1) Patients with second-generation Swanson trapeziometacarpal joint prostheses  were implanted in the hands | 1) Revision rate 2) Pain 3) Grip strength  4) Radiographic results | “The main problem associated with the prosthesis was dislocation. Surgical measures to improve  stability did not prevent this complication. The results after revision because of dislocation were no better than those associated with unrevised dislocated implants. In addition to dislocation, radiographic signs of silicone induced synovitis were frequently noted, although they did not necessarily lead to a poor result. We concluded that the results after long-term follow-up of the Swanson silicone trapezium implant for the treatment of primary osteoarthritis was poor and that our decision to stop using this implant in 1991 was correct.” |
| **Lovell et al, 1999** | Swanson trapezium implant | Interposition Complete Trapezial Replacement | NS | III | NS | 62 | 1) All patients treated by trapezial excision surgery for osteoarthritis | 1) Pain 2) Revision rate | “We conclude that trapeziectomy combined with Swanson implant gives better results in the short term if there are no complications of the operation.” |
|  | Sling excision |  |  |  |  |  |  |  |  |
| **Lehmann et al, 1998** | Swanson trapezium implant | Interposition Complete Trapezial Replacement | 27 | III | 65.4 | 67 | 1) Patients with tendon suspension interposition arthroplasty were compared with  2) Patients with prosthetic replacement of the trapezium for CMC I osteoarthritis | 1) Motion 2) Strength 3) Complications 4) Pain | “There might be a place for the condylar type of implants in low-demand rheumatoid patients, in whom as much bone stock  as possible should be preserved.” |
|  | Tendon group |  | 75 |  | 64.8 | 34 |  |  |  |
| **Lanzetta and Foucher, 1995** | Swanson trapezium implant | Interposition Complete Trapezial Replacement | NS | III | 56 | 60 | 1) Patients with osteoarthrosis of the CM joint of the thumb | 1) Grip Strength 2) Tip pinch 3) Pain | “The Ashworth-Blatt hemiarthroplasty failed to gain satisfactory results, but both silicone arthroplasty and soft tissue arthroplasty proved to be useful procedures. However, due to the risk of synovitis the present treatment of choice is soft tissue arthroplasty” |
|  | Ashworth-Blatt hemiarthroplasties |  |  |  |  |  |  |  |  |
|  | soft tissue arthroplasties |  |  |  |  |  |  |  |  |
| **Freeman and Homer, 1992** | Swanson trapezium implant | Interposition Complete Trapezial Replacement | 37 | IV | 60.5 | 66 | 1) Patients with Silastic trapezial arthroplasties | 1) Radiographic measurements 2) Complications  3) Pain relief | “We do not recommend this operation in young or particularly active patients, since we feel that tendon interposition arthroplasty provides a more durable result.” |
| **Creighton et al, 1991** | Swanson trapezium implant | Interposition Complete Trapezial Replacement | 124 | IV | 62 | 51 | 1) Patients with Silastic trapezial arthroplasties | 1) Radiographic measurements | “We conclude:  1. Specific postimplant radiographic changes were  seen in the areas of direct implant contact, 66% of the scaphoids and 74% of the first metacarpals.  2. Radiographic carpal and metacarpal changes were not found to be directly influenced by postoperative time.  3. Implant pinning did not influence the development of intracarpal cysts.  4. Despite obvious radiographic changes, patients with 84% (127 of 15 1) of the implants report complete satisfaction with the operation performed.  5. Systematic radiographic examination of the areas of implant contact within a uniform sample population with the use of a grading system has enabled better evaluation of long-term durability of Silastic trapezial arthroplasties, and in so doing, allows for data analysis relative to implant durability because it may relate to  the clinical problem of silicone synovitis.” |
| **Sollerman et al, 1988** | Swanson trapezium implant | Interposition Complete Trapezial Replacement | 33 | IV | 58 | 144 | 1) Patients with Swanson silastic trapezium implants | 1) Motion 2) Strength 3) Radiographic measurements 4) Complications | “The surgeon and patient must both be aware that silastic arthroplasty may not last a life-time and that revision surgery may be necessary if complications occur” |
| **Hay et al, 1988** | Swanson trapezium implant | Interposition Complete Trapezial Replacement | 52 | IV | 58 | 52.8 | 1) Patients with the Swanson implant arthroplasty for arthritis of the basal joint of the thumb | 1) Functional status 2) ROM 3) Key pinch 4) Complications | “Although we have documented no symptomatic cases of silicone synovitis and have not revised a prosthesis for this condition, we believe that prosthetic resorption, metacarpal resorption, and cyst formation indicate some degree of silicone synovitis that may eventually require revision surgery”. |
| **Amadio et al, 1982** | Swanson trapezium implant | Interposition Complete Trapezial Replacement | 21 | III | 60 | 26 | 1) All patients had trapeziometacarpal osteoarthritis. | 1) Pain relief 2) Functional status 3) ROM 4) Key pinch 5) Complications | “In this patient population, trapeziectomies with or without silicone replacement as treatment for trapeziometacarpal osteoarthritis appear to give similar results” |
|  | No implant |  | 24 |  | 54 | 37 |  |  |  |
| **Lister et al, 1977,** | Swanson trapezium implant | Interposition Complete Trapezial Replacement | 31 | IV | NS | 27.4 | 1) Patients with trapezial replacements | 1) Pain 2) Complications | “The procedure is not without complications. The three patients with radial neuritis serve to emphasize the great care which must be taken to preserve and gently retract the radial nerve which, if damaged in any way, produces such significant disability”. |
| **Gudmundsson et al, 1985** | Swanson trapezium implant | Interposition Complete Trapezial Replacement | 31 | IV | 59 | 78 | 1) Patients with pain due to carpometacarpal joint derangement | 1) Motion 2) Strength 3) Radiographic measurements | “The fact that pain relief, strength and stability were generally found to be satisfactory in spite of implant breakage, dislocation or incongruity in 20 of our 34 cases proves that although radiographically imperfect, they still functioned well as interposition plasties. The reactive fibrous tissue capsule around these implants probably is an important factor in preserving stability and freedom from pain” |
| **Ho et al, 1985** | Swanson trapezial implant, Eaton trapezial implant | Interposition Complete Trapezial Replacement | 25 | IV | 62 | 31 | 1) Arthroplasties with silicone rubber trapezium implants. | 1) Pain relief 2) Functional status 3) ROM 4) Key pinch | “In conclusion, we feel that the semi constrained trapezium implant provides a less mobile, but very stable CMC joint of the thumb. Our results for pain relief, functional improvement, and patient satisfaction are comparable with those reported for trapezium implant arthroplasty in the literature” |
| **Eaton, 1979** | Eaton trapezial implant | Trapezial Replacement | 46 | IV | 55 | 20.9 | 1) Patients have undergone replacement of the trapezium by the technique to be described with the perforated silicone-rubber implant. | 1) Complications 2) Pain relief 3) Grip and pinch strength | “The patients had intractable pain due to degenerative changes of multiple facets of the trapezium. The over-all rate of subluxation and dislocation was 10 per cent. In all hands, pain-free improved movement resulted. The pinch strength approximated the normal, averaging six kilograms for women and eight kilograms for men.” |
| **Nusem and Goodwin, 2003** | Gelfoam Spacer | Interposition Complete Trapezial Replacement | 30 | IV | 60 | 60 | 1) Interposition arthroplasties with Gelfoam were performed in patients between July 1990 and October 1996 2) All patients had symptomatic trapeziometacarpal osteoarthritis classified radiologically as Eaton stages 2–4 | 1) Pain 2) Patient satisfaction | “We conclude that excision of the trapezium and Gelfoam interposition has no specific complications and is a reliable surgical treatment for osteoarthritis of the trapeziometacarpal joint.” |
| **O’Leary et al, 2002** | Helal prosthesis | Interposition Complete Trapezial Replacement | 23 | IV | 63 | 59 | 1) Patients had a Helal prosthesis inserted following trapeziectomy for trapeziometacarpal arthritis. | 1) Pain relief 2) Grip and pinch strength | “We believe that the Helal silicone rubber ball spacer is a safe and reliable prosthesis for use in advanced trapeziometacarpal arthritis”. |
|  | Control |  | 19 |  | NS |  |  |  |  |
| **Grange and Helal, 1983** | Helal prosthesis | Interposition Complete Trapezial Replacement | 22 | IV | 56.8 | 13..6 | 1) Replacement of the trapezium of such cases with a silicone rubber spacer. | 1) Complications 2) Pain relief 3) Grip and pinch strength | “Other complications such as tender scars and nerve damage are largely avoidable with more care in technique, but we are satisfied that this surgical approach is satisfactory in our hands. The prosthesis would appear to perform at least as well as any so far devised and at a fraction of the cost.” |
| **Helal and McPherson, 1989** | Helal prosthesis | Interposition Complete Trapezial Replacement | 31 | IV | 55.4 | 32 | 1) Replacements were performed for osteoarthritis and for rheumatoid arthritis. | 1) Pain 2) Overall satisfaction 3) Standard measurements | “We conclude that the use of a bistemmed silicone elastomer ball spacer following trapeziectomy can achieve:  1. Reliable pain relief. 2. Maintenance of acceptable grip and pinch power. 3. Minimal loss of movement.” |
| **Greenberg et al, 1997** | Gore-Tex | Interposition Complete Trapezial Replacement | 31 | IV | 57 | 42 | 1) Symptomatic basilar thumb osteoarthritis (Eaton stages II-IV) with ePTFE interpositional arthroplasty | 1) Pain 2) Overall satisfaction 3) Standard measurements | “The authors recommend that use of this material for trapeziometacarpal or pan trapezial arthroplasty be abandoned.” |
| **Sotereanos et al, 1993** | Niebauer implant | Interposition Complete Trapezial Replacement | 27 | IV | 61 | 108 | 1) Patients with Preoperative trapeziometacarpal (TMC) joints were stage III or IV radiographically according to Eaton’s classification.5 | 1) Key pinch 2) Tip pinch 3) Grip strength 4) Radial abduction. | “We believe that the Niebauer trapeziometacarpal arthroplasty is a worthwhile procedure with a rare incidence of particulate synovitis. In 24 of 27 patients, pain was relieved and satisfactory motion and stability were achieved.” |
| **Sollerman et al, 1993** | Tecoflex polyurethane implant | Interposition Complete Trapezial Replacement | 25 | IV | 61 | 36 | 1) Patients with arthrosis of the first carpometacarpal joint were operated on.  2) Patients were selected if they consented to participate in the study | 1) Pain 2) ROM 3) Strength | “We conclude that polyurethane might be a suitable material for joint replacement, but such implants are not autoclavable and should be sterilized by gamma radiation. Polyurethane implants should be fixed to surrounding tissues by proper stabilizing  techniques because of the lack of fibrous encapsulation. The long-term results of polyurethane joint  replacement remain to be shown, but the mechanical properties of polyurethane and the short-term results indicate that problems of wear and unfavorable tissue reactions are less than after silicone replacement.” |
| **Kokkalis et al, 2009** | Acellular dermal allograft (GraftJacket) | Interposition Complete Trapezial Replacement | 89 | IV | 57 | 30 | 1) Primary surgery for thumb CMC joint osteoarthritis  2) A minimum of 12 months of follow-up. | 1) Pain levels 2) Grip strength 3) Key pinch strength  4) Range of motion 5) Radiographic measurements | “Trapeziectomy with suspension and interposition arthroplasty using an acellular dermal allograft is highly effective for the treatment of thumb carpometacarpal arthritis. This procedure provides a safe and effective alternative to autograft for both ligament reconstruction and tendon interposition and eliminates the potential morbidity of autograft harvest.” |
| **Marks et al 2017** | Acellular dermal allograft (GraftJacket) | Interposition Complete Trapezial Replacement | 31 | I | 65 | 12 | 1) They were diagnosed with CMC I OA and met indications for trapeziectomy with suspension interposition arthroplasty. | 1) MHQ  2)The Disabilities of the Arm, Shoulder, and Hand questionnaire (DASH) | “The use of the FCR tendon or allograft for trapeziectomy with suspension interposition arthroplasty in patients with CMC I OA leads to similar outcomes with more complications, mainly tendon irritations, associated with the latter. Therefore, we only use the  allograft in cases of severe instability requiring a larger amount of suspension-interposition  material or for revision procedures after failed suspension-interposition with the FCR tendon.” |
|  | FCR group |  | 29 |  | 64 |  |  |  |  |
| **Logli et al, 2017** | Acellular dermal allograft (GraftJacket) | Interposition Complete Trapezial Replacement | 30 | IV | 58.8 | 60 | 1) Patients with symptomatic isolated trapeziometacarpal arthritis, Eaton-Littler stages II and III | 1) NPRS 2) Quick Disabilities  of the Arm, Shoulder, and Hand (QuickDASH) 3) ROM | “An APT with interposition arthroplasty utilizing an acellular dermal matrix as the interposition material is a safe and reliable procedure with satisfactory outcomes at short- and long-term follow-up. Pain, strength, QuickDASH, and ROM do not significantly change between the 6-month and the 5-year follow-up.” |
| **Cobb et al, 2015** | Acellular dermal allograft  (GraftJacket)  and (OrthADAPT) | Interposition Complete Trapezial Replacement | 52 | IV | 59 | 88.8 | 1) Patients who underwent ARA for thumb carpometacarpal (CMC) with or without scaphotrapeziotrapezoid (STT) osteoarthritis (OA) between 2004 and 2011. | 1) Pain  2) Satisfaction  3) Grip Strength  4)Complications | “This study suggested that interposition is not necessary following ARA for thumb basal joint arthritis. Because arthroscopic interposition of material contributes to health care costs in terms of patient and facility costs without clear benefit to the patient, routine use of expensive interposition products should be abandoned or carefully evaluated with a prospective randomized controlled trial.” |
|  |  |  |  |  |  |  |  |  |  |
|  |  | Complete Trapezial Replacement without interposition | 73 |  | 52 | 67.2 |  |  |  |

***References from 41 – 143 can be found in Appedix 6**
