# Supplementary material for: Efficacy and Safety of Different Trapezium Implants for Trapeziometacarpal Joint Osteoarthritis: A Systematic Review and Meta-Analysis
Source: Hand (N Y). 2023 Jul 2;19(8):1242–51. doi: 10.1177/15589447231183172 (PMC11612267; doi:10.1177/15589447231183172)
Supplement: sj-docx-3-han-10.1177_15589447231183172 – Supplemental material for Efficacy and Safety of Different Trapezium Implants for Trapeziometacarpal Joint Osteoarthritis: A Systematic Review and Meta-Analysis [file sj-docx-3-han-10.1177_15589447231183172.docx]

**Supplementary Table 2:** NIH Quality Assessment Tool for Observational Cohort and Cross-Sectional Studies

| **Study ID** | **1. Was the research question or objective in this paper clearly stated?** | **2. Was the study population clearly specified and defined?** | **3. Was the participation rate of eligible persons at least 50%?** | **4. Were all the subjects selected or recruited from the same or similar populations (including the same time period)? Were inclusion and exclusion criteria for being in the study prespecified and applied uniformly to all participants?** | **5. Was a sample size justification, power description, or variance and effect estimates provided?** | **6. For the analyses in this paper, were the exposure(s) of interest measured prior to the outcome(s) being measured?** | **7. Was the time frame sufficient so that one could reasonably expect to see an association between exposure and outcome if it existed?** | **8. For exposures that can vary in amount or level, did the study examine different levels of the exposure as related to the outcome (e.g., categories of exposure, or exposure measured as continuous variable)?** | **9. Were the exposure measures (independent variables) clearly defined, valid, reliable, and implemented consistently across all study participants?** | **10. Was the exposure(s) assessed more than once over time?** | **11. Were the outcome measures (dependent variables) clearly defined, valid, reliable, and implemented consistently across all study participants?** | **12. Were the outcome assessors blinded to the exposure status of participants?** | **13. Was loss to follow-up after baseline 20% or less?** | **14. Were key potential confounding variables measured and adjusted statistically for their impact on the relationship between exposure(s) and outcome(s)?** | **Total scores** | **Overall quality** |
| --- | --- | --- | --- | --- | --- | --- | --- | --- | --- | --- | --- | --- | --- | --- | --- | --- |
|  | **Yes / No / Not reported (NR) or cannot determine (CD) or not applicable (NA)** | **Yes / No / Not reported (NR) or cannot determine (CD) or not applicable (NA)** | **Yes / No / Not reported (NR) or cannot determine (CD) or not applicable (NA)** | **Yes / No / Not reported (NR) or cannot determine (CD) or not applicable (NA)** | **Yes / No / Not reported (NR) or cannot determine (CD) or not applicable (NA)** | **Yes / No / Not reported (NR) or cannot determine (CD) or not applicable (NA)** | **Yes / No / Not reported (NR) or cannot determine (CD) or not applicable (NA)** | **Yes / No / Not reported (NR) or cannot determine (CD) or not applicable (NA)** | **Yes / No / Not reported (NR) or cannot determine (CD) or not applicable (NA)** | **Yes / No / Not reported (NR) or cannot determine (CD) or not applicable (NA)** | **Yes / No / Not reported (NR) or cannot determine (CD) or not applicable (NA)** | **Yes / No / Not reported (NR) or cannot determine (CD) or not applicable (NA)** | **Yes / No / Not reported (NR) or cannot determine (CD) or not applicable (NA)** | **Yes / No / Not reported (NR) or cannot determine (CD) or not applicable (NA)** |  |  |
| **Wachtl et al, 1998** | Yes | Yes | NR | NR | No | Yes | Yes | NA | NA | NA | Yes | NR | Yes | NR | 6.5 | **poor** |
| **Bricout and Rezzouk, 2016** | Yes | Yes | NR | NR | No | Yes | Yes | NA | NA | NA | NR | NR | Yes | NR | 5.5 | **poor** |
| **Lemoine et al, 2009** | Yes | Yes | NR | NR | No | Yes | Yes | NA | NA | NA | Yes | NR | Yes | NR | 6.5 | **poor** |
| **Nusem and Goodwin, 2003** | Yes | Yes | NR | NR | No | Yes | Yes | NA | NA | NA | NR | NR | Yes | NR | 5.5 | **poor** |
| **Robles-Molina et al, 2017** | Yes | Yes | NR | NR | No | Yes | Yes | NA | NA | NA | NR | NR | Yes | NR | 5.5 | **poor** |
| **Johnston et al, 2012** | Yes | Yes | NR | NR | No | Yes | Yes | NA | NA | NA | Yes | NR | Yes | NR | 6.5 | **poor** |
| **De Smet and Sioen, 2007** | Yes | Yes | NR | NR | No | Yes | Yes | NA | NA | NA | Yes | Yes | NR | NR | 6.5 | **poor** |
| **Sotereanos et al, 1993** | Yes | Yes | NR | NR | No | Yes | Yes | NA | NA | NA | NR | NR | NR | NR | 4.5 | **poor** |
| **Helal and McPherson, 1989** | Yes | Yes | NR | NR | No | Yes | Yes | NA | NA | NA | NR | NR | Yes | NR | 5.5 | **poor** |
| **Amadio et al, 1982** | Yes | Yes | NR | NR | No | Yes | Yes | NA | NA | NA | NR | NR | Yes | NR | 5.5 | **poor** |
| **Hay et al, 1988** | Yes | Yes | Yes | NR | No | Yes | Yes | NA | NA | NA | NA | NR | Yes | NR | 6.5 | **poor** |
| **Sollerman et al, 1988** | Yes | Yes | No | NR | No | Yes | Yes | NA | NA | NA | Yes | NR | No | NR | 6.5 | **poor** |
| **Creighton et al, 1991** | Yes | Yes | NR | NR | No | Yes | Yes | NA | NA | NA | Yes | NR | Yes | NR | 6.5 | **poor** |
| **Freeman and Homer, 1992** | Yes | Yes | No | NR | No | Yes | Yes | NA | NA | NA | Yes | NR | No | NR | 6.5 | **poor** |
| **Lehmann et al, 1998** | Yes | Yes | NR | NR | No | Yes | Yes | NA | NA | NA | Yes | Yes | Yes | NR | 7.5 | **fair** |
| **Lovell et al, 1999** | Yes | Yes | NR | Yes | No | Yes | Yes | NA | NA | NA | NR | NR | Yes | NR | 6.5 | **poor** |
| **Bezwada et al, 2002** | Yes | Yes | NR | NR | No | Yes | Yes | NA | NA | NA | Yes | NR | No | NR | 6 | **poor** |
| **MacDermid et al, 2003** | Yes | Yes | NR | NR | No | Yes | Yes | NA | NA | NA | Yes | NR | No | NR | 6 | **poor** |
| **Taylor et al, 2005** | Yes | Yes | NR | NR | No | Yes | Yes | NA | NA | NA | Yes | NR | Yes | NR | 6.5 | **poor** |
| **Jewell et al, 2011** | Yes | Yes | NR | NR | No | Yes | Yes | NA | NA | NA | Yes | NR | No | NR | 6 | **poor** |
| **Spaans et al, 2014** | Yes | Yes | NR | NR | No | Yes | Yes | NA | NA | NA | NR | NR | Yes | NR | 5.5 | **poor** |
| **Lister et al, 1977** | Yes | Yes | NR | NR | No | Yes | Yes | NA | NA | NA | NR | NR | Yes | NR | 5.5 | **poor** |
| **Van Aaken et al, 2016** | Yes | Yes | NR | Yes | No | Yes | Yes | NA | NA | NA | Yes | NR | Yes | NR | 7.5 | **fair** |
| **Colegate-Stone et al, 2011** | Yes | Yes | NR | NR | No | Yes | Yes | NA | NA | NA | Yes | NR | Yes | NR | 6.5 | **poor** |
| **Lanzetta and Foucher, 1995** | Yes | Yes | NR | NR | No | Yes | Yes | NA | NA | NA | Yes | NR | Yes | NR | 6 | **poor** |
| **Bell et al, 2011** | Yes | Yes | NR | Yes | No | Yes | Yes | NA | NA | NA | NR | NR | No | NR | 6 | **poor** |
| **Clarke et al, 2011** | Yes | Yes | NR | NR | No | Yes | Yes | NA | NA | NA | Yes | NR | Yes | NR | 6.5 | **poor** |
| **Blount et al, 2013** | Yes | Yes | NR | Yes | No | Yes | Yes | NA | NA | NA | Yes | Yes | Yes | NR | 8.5 | **fair** |
| **Odella et al, 2014** | Yes | Yes | NR | NR | No | Yes | Yes | NA | NA | NA | NR | NR | Yes | NR | 5.5 | **poor** |
| **Avisar et al, 2015** | Yes | Yes | NR | NR | No | Yes | Yes | NA | NA | NA | Yes | NR | Yes | NR | 6.5 | **poor** |
| **Jennings and Livingstone, 1990** | Yes | Yes | NR | NR | No | Yes | Yes | NA | NA | NA | NR | NR | Yes | NR | 5.5 | **poor** |
| **Conolly and Lanzetta, 1993** | Yes | Yes | NR | NR | No | Yes | Yes | NA | NA | NA | NR | NR | Yes | NR | 5.5 | **poor** |
| **Naidu et al, 2006** | Yes | Yes | NR | NR | No | Yes | Yes | NA | NA | NA | Yes | NR | Yes | NR | 6.5 | **poor** |
| **Martinez de Aragon et al, 2009** | Yes | Yes | NR | Yes | No | Yes | Yes | NA | NA | NA | Yes | NR | Yes | NR | 7.5 | **fair** |
| **Stillwater, 2017** | Yes | Yes | NR | Yes | No | Yes | Yes | NA | NA | NA | NR | NR | Yes | NR | 6.5 | **poor** |
| **Ferrari and Steffee, 1986** | Yes | Yes | NR | NR | No | Yes | Yes | NA | NA | NA | NR | NR | Yes | NR | 5.5 | **poor** |
| **Badia, 2006** | Yes | Yes | NR | NR | No | Yes | Yes | NA | NA | NA | NR | NR | Yes | NR | 5.5 | **poor** |
| **Toffoli and Teissier, 2017** | Yes | Yes | NR | NR | No | Yes | Yes | NA | NA | NA | Yes | NR | Yes | NR | 6.5 | **poor** |
| **Pendse et al, 2009** | Yes | Yes | NR | Yes | No | Yes | Yes | NA | NA | NA | Yes | NR | Yes | NR | 7.5 | **fair** |
| **Regnard, 2006** | Yes | Yes | NR | NR | No | Yes | Yes | NA | NA | NA | Yes | NR | Yes | NR | 6.5 | **poor** |
| **Ulrich-Vinther et al, 2008** | Yes | Yes | NR | Yes | No | Yes | Yes | NA | NA | NA | Yes | Yes | Yes | NR | 7.5 | **fair** |
| **Hansen et al, 2013** | Yes | Yes | NR | NR | No | Yes | Yes | NA | NA | NA | Yes | NR | No | NR | 6 | **poor** |
| **Thillemann et al, 2016** | Yes | Yes | NR | NR | No | Yes | Yes | NA | NA | NA | NR | NR | Yes | NR | 5.5 | **poor** |
| **Zollinger et al, 2008** | Yes | Yes | NR | NR | No | Yes | Yes | NA | NA | NA | Yes | NR | Yes | NR | 6.5 | **poor** |
| **Semere et al, 2015** | Yes | Yes | Yes | Yes | No | Yes | Yes | NA | NA | NA | NR | NR | Yes | NR | 7.5 | **fair** |
| **Eecken et al, 2012** | Yes | Yes | NR | NR | No | Yes | Yes | NA | NA | NA | NR | NR | Yes | NR | 5.5 | **poor** |
| **Spaans et al, 2016** | Yes | Yes | NR | NR | No | Yes | Yes | NA | NA | NA | Yes | NR | Yes | NR | 6.5 | **poor** |
| **August et al, 1984** | Yes | No | NR | NR | No | Yes | Yes | NA | NA | NA | NR | NR | Yes | NR | 5 | **poor** |
| **van Laarhoven et al, 2020** | Yes | Yes | NR | Yes | No | Yes | Yes | NA | NA | NA | Yes | NR | Yes | NR | 7.5 | **fair** |
| **Smeraglia et al, 2020** | Yes | Yes | NR | NR | No | Yes | Yes | NA | NA | NA | Yes | NR | Yes | NR | 6.5 | **poor** |
| **Sander et al, 2020** | Yes | Yes | NR | Yes | No | Yes | Yes | NA | NA | NA | Yes | NR | Yes | NR | 7.5 | **fair** |
| **Oh et al, 2019** | Yes | Yes | NR | Yes | Yes | Yes | Yes | NA | NA | NA | Yes | NR | Yes | NR | 7.5 | **fair** |
| **Mosegaard et al, 2020** | Yes | Yes | NR | Yes | No | Yes | Yes | NA | NA | NA | Yes | NR | Yes | Yes | 7.5 | **fair** |
| **Mattila et al, 2021** | Yes | Yes | NR | Yes | No | Yes | Yes | NA | NA | NA | Yes | NR | Yes | NR | 7.5 | **fair** |
| **Mattila et al, 2019** | Yes | Yes | NR | NR | No | Yes | Yes | NA | NA | NA | Yes | NR | Yes | NR | 6.5 | **poor** |
| **Martins et al, 2020** | Yes | Yes | NR | NR | No | Yes | Yes | NA | NA | NA | Yes | NR | Yes | NR | 6.5 | **poor** |
| **Martin-Ferrero et al, 2021** | Yes | Yes | NR | Yes | No | Yes | Yes | NA | NA | NA | Yes | NR | Yes | NR | 7.5 | **fair** |
| **Logli et al, 2017** | Yes | Yes | NR | NR | No | Yes | Yes | NA | NA | NA | Yes | NR | Yes | NR | 6.5 | **poor** |
| **Logan et al,2020** | Yes | Yes | NR | NR | No | Yes | Yes | NA | NA | NA | Yes | NR | Yes | NR | 6.5 | **poor** |
| **Lallemand et al, 2019** | Yes | Yes | NR | NR | No | Yes | Yes | NA | NA | NA | NR | NR | Yes | NR | 5.5 | **poor** |
| **Kirkeby et al, 2021** | Yes | Yes | NR | NR | No | Yes | Yes | NA | NA | NA | Yes | NR | Yes | NR | 6.5 | **poor** |
| **Kennedy et al,2019** | Yes | Yes | NR | Yes | No | Yes | Yes | NA | NA | NA | Yes | NR | Yes | NR | 7.5 | **fair** |
| **gomez-garrido 2019** | Yes | Yes | NR | NR | No | Yes | Yes | NA | NA | NA | Yes | NR | Yes | NR | 6.5 | **poor** |
| **Froschauer et al, 2020** | Yes | Yes | NR | Yes | No | Yes | Yes | NA | NA | NA | Yes | NR | Yes | NR | 7.5 | **fair** |
| **Froschauer et al,2019** | Yes | Yes | NR | Yes | No | Yes | Yes | NA | NA | NA | Yes | NR | Yes | NR | 7.5 | **fair** |
| **Gonzalez-Espino et al, 2021** | Yes | Yes | NR | Yes | No | Yes | Yes | NA | NA | NA | Yes | NR | Yes | NR | 7.5 | **fair** |
| **Erne et al, 2018** | Yes | Yes | NR | Yes | No | Yes | Yes | NA | NA | NA | NR | NR | Yes | NR | 6.5 | **poor** |
| **Dumartinet-Gibaud et al, 2020** | Yes | Yes | NR | Yes | No | Yes | Yes | NA | NA | NA | Yes | NR | Yes | NR | 7.5 | **fair** |
| **Dreant et al, 2018** | Yes | Yes | NR | Yes | No | Yes | Yes | NA | NA | NA | Yes | NR | Yes | Yes | 8.5 | **fair** |
| **Dietrich et al, 2021** | Yes | Yes | NR | Yes | No | Yes | Yes | NA | NA | NA | Yes | NR | Yes | NR | 7.5 | **fair** |
| **Cebrian-Gomez et al,2018** | Yes | Yes | NR | Yes | No | Yes | Yes | NA | NA | NA | Yes | NR | Yes | NR | 7.5 | **fair** |
| **Caekebeke et al, 2017** | Yes | Yes | NR | NR | No | Yes | Yes | NA | NA | NA | Yes | NR | Yes | NR | 6.5 | **poor** |
| **Andrzejewski et al, 2019** | Yes | Yes | NR | NR | No | Yes | Yes | NA | NA | NA | Yes | NR | Yes | NR | 6.5 | **poor** |
| **Cobb et al, 2015** | Yes | Yes | NR | Yes | No | Yes | Yes | NA | NA | NA | Yes | NR | Yes | Yes | 8.5 | **fair** |
| **Craik et al, 2017** | Yes | Yes | NR | Yes | No | Yes | Yes | NA | NA | NA | Yes | NR | Yes | Yes | 8.5 | **fair** |
| **Adams et al, 2009** | Yes | Yes | NR | Yes | No | Yes | Yes | NA | NA | NA | Yes | NR | Yes | NR | 7.5 | **fair** |
| **Dehl et al, 2017** | Yes | Yes | No | Yes | No | Yes | Yes | NA | NA | NA | Yes | NR | No | NR | 7.5 | **fair** |
| **van Cappelle et al, 2001** | Yes | Yes | NR | Yes | No | Yes | Yes | NA | NA | NA | Yes | NR | Yes | NR | 7.5 | **fair** |
| **Mariconda et al, 2014** | Yes | Yes | NR | NR | No | Yes | Yes | NA | NA | NA | Yes | NR | Yes | NR | 6.5 | **poor** |
| **O’Leary et al, 2002** | Yes | Yes | NR | NR | No | Yes | Yes | NA | NA | NA | Yes | NR | Yes | NR | 6.5 | **poor** |
