# Supplementary material for: Efficacy and Safety of Different Trapezium Implants for Trapeziometacarpal Joint Osteoarthritis: A Systematic Review and Meta-Analysis
Source: Hand (N Y). 2023 Jul 2;19(8):1242–51. doi: 10.1177/15589447231183172 (PMC11612267; doi:10.1177/15589447231183172)
Supplement: sj-docx-4-han-10.1177_15589447231183172 – Supplemental material for Efficacy and Safety of Different Trapezium Implants for Trapeziometacarpal Joint Osteoarthritis: A Systematic Review and Meta-Analysis [file sj-docx-4-han-10.1177_15589447231183172.docx]

**Supplementary Table 3:** NIH Quality Assessment Tool for Single-arm studies

| **Study ID** | **1. Was the research question or objective in this paper clearly stated?** | **2. Were eligibility/selection criteria for the study population prespecified and clearly described?** | **3. Were the participants in the study representative of those who would be eligible for the test/service/intervention in the general or clinical population of interest?** | **4. Were all eligible participants that met the prespecified entry criteria enrolled?** | **5. Was the sample size sufficiently large to provide confidence in the findings?** | **6. Was the test/service/intervention clearly described and delivered consistently across the study population?** | **7. Were the outcome measures prespecified, clearly defined, valid, reliable, and assessed consistently across all study participants?** | **8. Were the people assessing the outcomes blinded to the participants' exposures/interventions?** | **9. Was the loss to follow-up after baseline 20% or less? Were those lost to follow-up accounted for in the analysis?** | **10. Did the statistical methods examine changes in outcome measures from before to after the intervention? Were statistical tests done that provided p values for the pre-to-post changes?** | **11. Were outcome measures of interest taken multiple times before the intervention and multiple times after the intervention (i.e., did they use an interrupted time-series design)?** | **12. If the intervention was conducted at a group level (e.g., a whole hospital, a community, etc.) did the statistical analysis take into account the use of individual-level data to determine effects at the group level?** | **Total scores** | **Overall quality** |
| --- | --- | --- | --- | --- | --- | --- | --- | --- | --- | --- | --- | --- | --- | --- |
|  | **Yes / No / Not reported (NR) or cannot determine (CD) or not applicable (NA)** | **Yes / No / Not reported (NR) or cannot determine (CD) or not applicable (NA)** | **Yes / No / Not reported (NR) or cannot determine (CD) or not applicable (NA)** | **Yes / No / Not reported (NR) or cannot determine (CD) or not applicable (NA)** | **Yes / No / Not reported (NR) or cannot determine (CD) or not applicable (NA)** | **Yes / No / Not reported (NR) or cannot determine (CD) or not applicable (NA)** | **Yes / No / Not reported (NR) or cannot determine (CD) or not applicable (NA)** | **Yes / No / Not reported (NR) or cannot determine (CD) or not applicable (NA)** | **Yes / No / Not reported (NR) or cannot determine (CD) or not applicable (NA)** | **Yes / No / Not reported (NR) or cannot determine (CD) or not applicable (NA)** | **Yes / No / Not reported (NR) or cannot determine (CD) or not applicable (NA)** | **Yes / No / Not reported (NR) or cannot determine (CD) or not applicable (NA)** |  |  |
| **Bengezi and Vo, 2014** | Yes | Yes | Yes | CD | No | Yes | No | No | Yes | No | No | NA | 7 | **Fair** |
| **Martin-Ferrero, 2014** | Yes | No | No | CD | No | Yes | Yes | NR | Yes | Yes | Yes | NA | 7.5 | **Fair** |
| **Sollerman et al., 1993** | Yes | Yes | No | CD | No | Yes | NR | NR | Yes | NR | No | NA | 5.5 | **Poor** |
| **Klahn et al., 2012** | Yes | Yes | Yes | CD | No | NR | Yes | NR | No | Yes | Yes | NA | 7 | **Fair** |
| **Engel et al.,**  **1982** | Yes | No | Yes | CD | No | Yes | Yes | NR | Yes | NR | No | NA | 6.5 | **Fair** |
| **Aita et al,**  **2016** | Yes | No | Yes | CD | No | Yes | NR | NR | Yes | NR | No | NA | 5.5 | **Fair** |
| **Chakrabarti et al, 1997** | Yes | Yes | Yes | CD | No | Yes | Yes | NR | NR | NR | Yes | NA | 6.5 | **Fair** |
| **Kokkalis et al., 2009** | Yes | Yes | Yes | CD | No | Yes | Yes | NR | Yes | Yes | No | NA | 8 | **Fair** |
| **Greenberg et al., 1997** | Yes | Yes | Yes | CD | No | Yes | Yes | NR | Yes | NR | No | NA | 7 | **Fair** |
| **Grange and Helal, 1983** | Yes | Yes | Yes | CD | No | Yes | No | NR | Yes | NR | No | NA | 6.5 | **Fair** |
| **Eaton,**  **1979** | Yes | Yes | Yes | CD | No | Yes | Yes | NR | Yes | NR | No | NA | 7 | **Fair** |
| **Ho et al.,**  **1985** | Yes | Yes | Yes | CD | No | Yes | Yes | NR | Yes | NR | No | NA | 7 | **Fair** |
| **Gudmundsson et al., 1985** | Yes | Yes | Yes | CD | No | Yes | NR | NR | Yes | NR | No | NA | 6 | **Poor** |
| **Szalay et al., 2013** | Yes | Yes | Yes | CD | No | Yes | Yes | NR | Yes | NR | No | NA | 7 | **Fair** |
| **About et al., 2016** | Yes | Yes | Yes | CD | No | Yes | Yes | NR | Yes | NR | No | NA | 7 | **Fair** |
| **Kessler et al., 1984** | Yes | Yes | Yes | CD | No | Yes | NR | NR | Yes | NR | No | NA | 6 | **Poor** |
| **Ashworth et al., 1977** | Yes | Yes | Yes | CD | No | Yes | NR | NR | Yes | NR | No | NA | 6 | **Poor** |
| **Diacom et al., 2011** | Yes | Yes | Yes | CD | No | Yes | Yes | NR | Yes | Yes | No | NA | 8 | **Poor** |
| **Russo et al., 2016** | Yes | Yes | NR | CD | No | Yes | NR | NR | Yes | Yes | No | NA | 6 | **Poor** |
| **Swanson et al., 1981** | Yes | Yes | Yes | CD | No | Yes | NR | NR | Yes | NR | No | NA | 6 | **Poor** |
| **Howard et al., 1985** | Yes | Yes | Yes | CD | No | Yes | NR | NR | Yes | NR | No | NA | 6 | **Poor** |
| **Pritchett and Gabriel, 2012** | Yes | Yes | Yes | CD | No | Yes | Yes | NR | Yes | NR | Yes | NA | 7.5 | **Fair** |
| **Cooney et al., 1987** | Yes | Yes | NR | CD | No | Yes | NR | NR | Yes | NR | No | NA | 5 | **Poor** |
| **Hannula and Nahigian, 1999** | Yes | Yes | NR | CD | No | Yes | NR | NR | Yes | Yes | No | NA | 6 | **Poor** |
| **Kollig et al., 2017** | Yes | Yes | Yes | CD | No | Yes | NR | NR | CD | NR | No | NA | 5 | **Poor** |
| **Goubau et al, 2013** | Yes | Yes | Yes | CD | No | Yes | Yes | NR | Yes | Yes | Yes | NA | 8.5 | **Fair** |
| **De la Caffinière and Aucouturier** | Yes | Yes | NR | CD | No | Yes | NR | NR | Yes | NR | CD | NA | 4.5 | **Poor** |
| **Boeckstyns et al., 1989** | Yes | Yes | Yes | CD | No | Yes | NR | NR | Yes | No | Yes | NA | 7 | **Fair** |
| **Sondergaard et al., 1991** | Yes | Yes | Yes | CD | No | Yes | Yes | NR | Yes | No | Yes | NA | 8 | **Fair** |
| **Van Cappelle et al, 1999** | Yes | Yes | Yes | CD | No | Yes | Yes | NR | Yes | No | Yes | NA | 8 | **Fair** |
| **Vissers et al., 2018** | Yes | Yes | Yes | CD | No | Yes | Yes | NR | Yes | Yes | No | NA | 8 | **Fair** |
| **Tchurukdichian et al., 2021** | Yes | Yes | Yes | CD | No | Yes | Yes | NR | Yes | Yes | Yes | NA | 8.5 | **Fair** |
| **Tchurukdichian et al., 2020** | Yes | Yes | Yes | CD | No | Yes | Yes | NR | Yes | Yes | Yes | NA | 8.5 | **Fair** |
| **Mattila et al, 2017** | Yes | Yes | NR | CD | No | Yes | Yes | NR | Yes | Yes | Yes | NA | 7.5 | **Fair** |
| **Martin-Ferrero et al, 2019** | Yes | Yes | Yes | CD | No | Yes | Yes | NR | Yes | Yes | No | NA | 8 | **Fair** |
| **Lussiez et al, 2021** | Yes | Yes | Yes | CD | No | Yes | Yes | NR | Yes | NR | Yes | NA | 7.5 | **Fair** |
| **Gerace et al., 2020** | Yes | Yes | Yes | No | No | Yes | Yes | NR | Yes | Yes | Yes | NA | 9 | **Fair** |
| **Froschauer et al, 2021** | Yes | Yes | Yes | CD | No | Yes | Yes | NR | Yes | Yes | Yes | NA | 8.5 | **Fair** |
| **Dremstrup et al., 2020** | Yes | Yes | No | CD | No | Yes | Yes | NR | Yes | Yes | Yes | NA | 8 | **Fair** |
| **De Smet et al., 2020** | Yes | Yes | Yes | CD | No | Yes | Yes | NR | Yes | No | Yes | NA | 8 | **Fair** |
