# Supplementary material for: Efficacy and Safety of Different Trapezium Implants for Trapeziometacarpal Joint Osteoarthritis: A Systematic Review and Meta-Analysis
Source: Hand (N Y). 2023 Jul 2;19(8):1242–51. doi: 10.1177/15589447231183172 (PMC11612267; doi:10.1177/15589447231183172)
Supplement: sj-docx-5-han-10.1177_15589447231183172 – Supplemental material for Efficacy and Safety of Different Trapezium Implants for Trapeziometacarpal Joint Osteoarthritis: A Systematic Review and Meta-Analysis [file sj-docx-5-han-10.1177_15589447231183172.docx]

**Supplementary Table 4:** The Cochrane Collaboration’s tool for assessing risk of bias

| **Study ID** | **Random sequence generation (selection bias)** | | **Allocation concealment (selection bias)** | | **Blinding of participants and personnel (performance bias)** | | **Blinding of outcome assessment (detection bias)** | | **Incomplete outcome data (attrition bias)** | | **Selective reporting (reporting bias)** | | **Other Bias** | |
| --- | --- | --- | --- | --- | --- | --- | --- | --- | --- | --- | --- | --- | --- | --- |
|  | Low/ High/ Unclear risk of bias | Reason | Low/High/ Unclear risk of bias | Reason | Low/High/ Unclear risk of bias | Reason | Low/High/ Unclear risk of bias | Reason | Low/ High/ Unclear risk of bias | Reason | Low/High/ Unclear risk of bias | Reason | Low/ High/ Unclear risk of bias | Reason |
|  |  |  |  |  |  |  |  |  |  |  |  |  |  |  |
| **Hansen and stilling, 2013** | Low risk of bias | “Randomization was done by drawing labels from a box, and the labels were then concealed in 32 sequentially numbered closed envelopes” | Low risk of bias | “Randomization was done by drawing labels from a box, and the labels were then concealed in 32 sequentially numbered closed envelopes” | Low risk of bias | "Patient-blinded clinical trial " | High risk of bias | “Only Patients were blinded” | Low risk of bias | No risk of attrition bias was observed. | Low risk of bias | The study has followed the protocol | Low risk of bias | No other sources of bias |
| **Nilsson et al, 2010** | Low risk of bias | “According to a randomization list and by using closed envelopes” | Low risk of bias | “According to a randomization list and by using closed envelopes” | High risk of bias | “Only observer was blinded” | Low risk of bias | “Observer was blinded” | Low risk of bias | No risk of attrition bias was observed. | unclear | study protocol not reported | High risk of bias | "The study was supported by Artimplant AB, Göteborg, Sweden. The sponsor monitored the study, collected and analyzed the data, and gave support for the manuscript." |
| **Mark et al, 2017** | Low risk of bias | "A computer-generated random-numbers table was used and sealed opaque envelopes containing the corresponding intervention were prepared." | Low risk of bias | "A computer-generated random-numbers table was used and sealed opaque envelopes containing the corresponding the intervention was prepared." | High risk of bias | Open-Label | Low risk of bias | "Evaluated by an independent physician who was blinded to the study protocol" | Low risk of bias | No risk of attrition bias was observed. | Low risk of bias | The study has followed the protocol | Low risk of bias | No other sources of bias |
