# Supplementary material for: Efficacy and Safety of Different Trapezium Implants for Trapeziometacarpal Joint Osteoarthritis: A Systematic Review and Meta-Analysis
Source: Hand (N Y). 2023 Jul 2;19(8):1242–51. doi: 10.1177/15589447231183172 (PMC11612267; doi:10.1177/15589447231183172)
Supplement: sj-docx-6-han-10.1177_15589447231183172 – Supplemental material for Efficacy and Safety of Different Trapezium Implants for Trapeziometacarpal Joint Osteoarthritis: A Systematic Review and Meta-Analysis [file sj-docx-6-han-10.1177_15589447231183172.docx]

**Appendix 6 – References of the included 123 studies**

Adams BD, Pomerance J, Nguyen A, Kuhl TL. Early outcome of spherical ceramic trapezial-metacarpal arthroplasty. *J Hand Surg Am*. Feb 2009;34(2):213-8. doi:10.1016/j.jhsa.2008.10.017.

Agout C, Ardouin L, Bellemere P. A ten-year prospective outcome study of Pi2 pyrocarbon spacer arthroplasty in carpometacarpal joint osteoarthritis. *Hand Surg Rehabil*. Sep 2016;35(4):255-261. doi:10.1016/j.hansur.2016.05.002.

Aita MA, Alves RS, Longuino LF, Ferreira CH, Ikeuti DH, Rodrigues LM. Measurement of quality of life among patient undergoing arthroplasty of the thumb to treat CMC arthritis. *Rev Bras Ortop*. Jul-Aug 2016;51(4):431-6. doi:10.1016/j.rboe.2016.06.003.

Amadio PC, Millender LH, Smith RJ. Silicone spacer or tendon spacer for trapezium resection arthroplasty—Comparison of results. *The Journal of Hand Surgery*. 1982/05 1982;7(3):237-244. doi:10.1016/s0363-5023(82)80173-1.

Andrzejewski A, Ledoux P. Maia((R)) trapeziometacarpal joint arthroplasty: Survival and clinical outcomes at 5 years' follow-up. *Hand Surg Rehabil*. Jun 2019;38(3):169-173. doi:10.1016/j.hansur.2019.03.004.

Ashworth CR, Blatt G, Chuinard RG, Stark HH. Silicone-rubber interposition arthroplasty of the carpometacarpal joint of the thumb. *The Journal of Hand Surgery*. 1977/09 1977;2(5):345-357. doi:10.1016/s0363-5023(77)80044-0.

August AC, Coupland RM, Sandifer JP. Short term review of the De La Caffiniere trapeziometacarpal arthroplasty. J Hand Surg Br. Jun 1984;9(2):185-8.

Avisar E, Elvey M, Tzang C, Sorene E. Trapeziectomy With a Tendon Tie-in Implant for Osteoarthritis of the Trapeziometacarpal Joint. *J Hand Surg Am*. Jul 2015;40(7):1292-7. doi:10.1016/j.jhsa.2015.04.020.

Badia A. Trapeziometacarpal arthroscopy: a classification and treatment algorithm. *Hand Clin*. May 2006;22(2):153-63. doi:10.1016/j.hcl.2006.02.006.

Bell R, Desai S, House H, O'Donovan T, Palmer AK. A retrospective multicenter study of the Artelon(R) carpometacarpal joint implant. *Hand (N Y)*. Dec 2011;6(4):364-72. doi:10.1007/s11552-011-9366-0.

Bengezi O, Vo A. Early outcomes of arthroplasty of the first carpometacarpal joint using pyrocarbon spherical implants. *Plastic surgery (Oakville, Ont)*. Summer 2014;22(2):79-82.

Bezwada HP, Sauer ST, Hankins ST, Webber JB. Long-term results of trapeziometacarpal silicone arthroplasty. *J Hand Surg Am*. May 2002;27(3):409-17. doi:10.1053/jhsu.2002.31733.

Blount AL, Armstrong SD, Yuan F, Burgess SD. Porous polyurethaneurea (Artelon) joint spacer compared to trapezium resection and ligament reconstruction. *J Hand Surg Am*. Sep 2013;38(9):1741-5. doi:10.1016/j.jhsa.2013.05.013.

Boeckstyns ME, Sinding A, Elholm KT, Rechnagel K. Replacement of the trapeziometacarpal joint with a cemented (Caffinière) prosthesis. J Hand Surg Am. Jan 1989;14(1):83-9. doi:10.1016/0363-5023(89)90063-4

Bricout M, Rezzouk J. Complications and failures of the trapeziometacarpal Maia((R)) prosthesis: A series of 156 cases. *Hand Surg Rehabil*. Jun 2016;35(3):190-198. doi:10.1016/j.hansur.2016.02.005.

Caekebeke P, Duerinckx J. Can surgical guidelines minimize complications after Maia(R) trapeziometacarpal joint arthroplasty with unconstrained cups? *J Hand Surg Eur Vol*. May 2018;43(4):420-425. doi:10.1177/1753193417741237.

Cebrian-Gomez R, Lizaur-Utrilla A, Sebastia-Forcada E, Lopez-Prats FA. Outcomes of cementless joint prosthesis versus tendon interposition for trapeziometacarpal osteoarthritis: a prospective study. *J Hand Surg Eur Vol*. Feb 2019;44(2):151-158. doi:10.1177/1753193418787151.

Chakrabati A, Robinson A, Gallagher PJJHSB. De la Caffiniere thumb carpometacarpal replacements. 1997;22:695-698.

Clarke S, Hagberg W, Kaufmann RA, Grand A, Wollstein R. Complications with the use of Artelon in thumb CMC joint arthritis. *Hand (N Y)*. Sep 2011;6(3):282-6. doi:10.1007/s11552-011-9332-x.

Cobb TK, Walden AL, Cao Y. Long-Term Outcome of Arthroscopic Resection Arthroplasty With or Without Interposition for Thumb Basal Joint Arthritis. *J Hand Surg Am*. Sep 2015;40(9):1844-51. doi:10.1016/j.jhsa.2015.05.010.

Colegate-Stone TJ, Garg S, Subramanian A, Mani GV. Outcome analysis of trapezectomy with and without pyrocarbon interposition to treat primary arthrosis of the trapeziometacarpal joint. *Hand Surg*. 2011;16(1):49-54. doi:10.1142/S0218810411005060.

Conolly WB, Lanzetta M. Surgical management of arthritis of the carpo-metacarpal joint of the thumb. The Australian and New Zealand journal of surgery. Aug 1993;63(8):596-603. doi:10.1111/j.1445-2197.1993.tb00465.x

Cooney WP, Linscheid RL, Askew LJ. Total arthroplasty of the thumb trapeziometacarpal joint. Clin Orthop Relat Res. Jul 1987;(220):35-45.

Craik JD, Glasgow S, Andren J, et al. Early Results of the ARPE Arthroplasty Versus Trapeziectomy for the Treatment of Thumb Carpometacarpal Joint Osteoarthritis. *J Hand Surg Asian Pac Vol*. Dec 2017;22(4):472-478. doi:10.1142/S0218810417500526.

Creighton Jr JJ, Steichen JB, Strickland JWJTJohs. Long-term evaluation of Silastic trapezial arthroplasty in patients with osteoarthritis. 1991;16(3):510-519.

de la Caffiniere JY, Aucouturier P. Trapezio-metacarpal arthroplasty by total prosthesis. The Hand. Feb 1979;11(1):41-6. doi:10.1016/s0072-968x(79)80007-8

De Smet A, Vanhove W, Benis S, Verstraete M, Hollevoet N. Ten-year outcomes of the Arpe prosthesis for the treatment of osteoarthritis of the trapeziometacarpal joint. *Acta orthopaedica Belgica*. Mar 2020;86(1):131-136.

De Smet L, Sioen W. Basal joint osteoarthritis of the thumb: trapeziectomy, with or without tendon interposition, or total joint arthroplasty? A prospective study. *European Journal of Orthopaedic Surgery & Traumatology*. 2007;17(5):431-436. doi:10.1007/s00590-007-0219-z.

Dehl M, Chelli M, Lippmann S, Benaissa S, Rotari V, Moughabghab M. Results of 115 Rubis II reverse thumb carpometacarpal joint prostheses with a mean follow-up of 10 years. *J Hand Surg Eur Vol*. Jul 2017;42(6):592-598. doi:10.1177/1753193416687508.

Diaconu M, Mathoulin C, Facca S, Liverneaux P. Arthroscopic interposition arthroplasty of the trapeziometacarpal joint. *Chir Main*. Sep 2011;30(4):282-7. doi:10.1016/j.main.2011.06.009.

Dietrich A, Winkelmann M, O'Loughlin PF, Krettek C, Gaulke R. Arthroplasty of the trapeziometacarpal joint with or without bioabsorbable polylactide scaffold (RegJoint) interposition. *Hand Surg Rehabil*. Feb 2022;41(1):65-72. doi:10.1016/j.hansur.2021.09.010.

Dreant N, Poumellec MA. Total Thumb Carpometacarpal Joint Arthroplasty: A Retrospective Functional Study of 28 MOOVIS Prostheses. *Hand (N Y)*. Jan 2019;14(1):59-65. doi:10.1177/1558944718797341.

Dremstrup L, Thillemann JK, Kirkeby L, Larsen LP, Hansen TB, Stilling M. Two-year results of the Moovis trapeziometacarpal joint arthroplasty with focus on early complications. *J Hand Surg Eur Vol*. Feb 2021;46(2):131-140. doi:10.1177/1753193420921307.

Dumartinet-Gibaud R, Bigorre N, Raimbeau G, Jeudy J, Saint Cast Y. Arpe total joint arthroplasty for trapeziometacarpal osteoarthritis: 80 thumbs in 63 patients with a minimum of 10 years follow-up. *J Hand Surg Eur Vol*. Jun 2020;45(5):465-469. doi:10.1177/1753193420909198.

Eaton RG. Replacement of the trapezium for arthritis of the basal articulations: a new technique with stabilization by tenodesis. The Journal of bone and joint surgery American volume. Jan 1979;61(1):76-82.

Eecken SV, Vanhove W, Hollevoet N. Trapeziometacarpal joint replacement with the Arpe prosthesis. *Acta orthopaedica Belgica*. Dec 2012;78(6):724-9.

Engel J, Ganel A, Patish H, Kamhin MJAOS. Osteoarthritis of the trapezio-metacarpal joint: Results of treatment with a silicone cap implant. 1982;53(2):219-223.

Erne H, Scheiber C, Schmauss D, et al. Total Endoprosthesis Versus Lundborg s Resection Arthroplasty for the Treatment of Trapeziometacarpal Joint Osteoarthritis. *Plast Reconstr Surg Glob Open*. Apr 2018;6(4):e1737. doi:10.1097/GOX.0000000000001737.

Ferrari B, Steffee AD. Trapeziometacarpal total joint replacement using the Steffee prosthesis. The Journal of bone and joint surgery American volume. Oct 1986;68(8):1177-84.

Freeman G, Honner RJJoHS. Silastic replacement of the trapezium. 1992;17(4):458-462.

Froschauer SM, Holzbauer M, Hager D, Schnelzer R, Kwasny O, Duscher D. Elektra prosthesis versus resection-suspension arthroplasty for thumb carpometacarpal osteoarthritis: a long-term cohort study. *J Hand Surg Eur Vol*. Jun 2020;45(5):452-457. doi:10.1177/1753193419873230.

Froschauer SM, Holzbauer M, Mihalic JA, Kwasny O. TOUCH((R)) Prosthesis for Thumb Carpometacarpal Joint Osteoarthritis: A Prospective Case Series. *J Clin Med*. Sep 10 2021;10(18)doi:10.3390/jcm10184090.

Froschauer SM, Holzbauer M, Schnelzer RF, et al. Total arthroplasty with Ivory((R)) prosthesis versus resection-suspension arthroplasty: a retrospective cohort study on 82 carpometacarpal-I osteoarthritis patients over 4 years. *Eur J Med Res*. Apr 15 2020;25(1):13. doi:10.1186/s40001-020-00411-8.

Gerace E, Royaux D, Gaisne E, Ardouin L, Bellemere P. Pyrocardan(R) implant arthroplasty for trapeziometacarpal osteoarthritis with a minimum follow-up of 5 years. *Hand Surg Rehabil*. Dec 2020;39(6):528-538. doi:10.1016/j.hansur.2020.09.003.

Gomez-Garrido D, Trivino-Mayoral V, Delgado-Alcala V, et al. Five year long term results of total joint arthroplasties in the treatment of trapeziometacarpal osteoarthritis. *Acta Biomed*. Dec 23 2019;90(4):451-456. doi:10.23750/abm.v90i4.8131.

Gonzalez-Espino P, Pottier M, Detrembleur C, Goffin D. Touch(R) double mobility arthroplasty for trapeziometacarpal osteoarthritis: outcomes for 92 prostheses. *Hand Surg Rehabil*. Dec 2021;40(6):760-764. doi:10.1016/j.hansur.2021.08.005.

Goubau JF, Goorens CK, Van Hoonacker P, Berghs B, Kerckhove D, Scheerlinck T. Clinical and radiological outcomes of the Ivory arthroplasty for trapeziometacarpal joint osteoarthritis with a minimum of 5 years of follow-up: a prospective single-centre cohort study. *J Hand Surg Eur Vol*. Oct 2013;38(8):866-74. doi:10.1177/1753193413488494.

Grange W, Helal BJH. Replacement of the trapezium with a silicone rubber ball spacer. 1983;(1):53-56.

Greenberg JA, Mosher Jr JF, Fatti JFJTJohs. X-ray changes after expanded polytetrafluoroethylene (Gore-Tex) interpositional arthroplasty. 1997;22(4):658-663.

Gudmundsson G, Jonsson E, Sigurdsson B. Trapezium replacement arthroplasty. *Acta Orthop Scand*. Jun 1985;56(3):245-8. doi:10.3109/17453678508993005.

Hannula TT, Nahigian SH. A preliminary report: cementless trapeziometacarpal arthroplasty. J Hand Surg Am. Jan 1999;24(1):92-101. doi:10.1053/jhsu.1999.jhsu24a0092.

Hansen TB, Dremstrup L, Stilling M. Patients with metal-on-metal articulation in trapeziometacarpal total joint arthroplasty may have elevated serum chrome and cobalt. *J Hand Surg Eur Vol*. Oct 2013;38(8):860-5. doi:10.1177/1753193413487685.

Hansen TB, Stilling M. Equally good fixation of cemented and uncemented cups in total trapeziometacarpal joint prostheses. A randomized clinical RSA study with 2-year follow-up. *Acta Orthop*. Feb 2013;84(1):98-105. doi:10.3109/17453674.2013.765625.

Hay EL, Bomberg BC, Burke C, Misenheimer CJTJoA. Long-term results of silicone trapezial implant arthroplasty. 1988;3(3):215-223.

Helal B, McPherson IJTJoHSB, Volume E. Replacement of the trapezium with a silicone elastomer universal small joint spacer. 1989;14(4):456-459.

Ho PK, Jacobs JL, Clark GL. Trapezium implant arthroplasty: Evaluation of a semiconstrained implant. *The Journal of Hand Surgery*. 1985/09 1985;10(5):654-660. doi:10.1016/s0363-5023(85)80202-1.

Howard FM, Simpson LA, Belsole RJ. Silastic condylar arthroplasty. Clin Orthop Relat Res. May 1985;(195):144-50.

Jennings CD, Livingstone DP. Convex condylar arthroplasty of the basal joint of the thumb: failure under load. J Hand Surg Am. Jul 1990;15(4):573-81. doi:10.1016/s0363-5023(09)90018-1

Jewell DP, Brewster MB, Arafa MA. Outcomes of silastic trapezium replacements. *Hand Surg*. 2011;16(3):301-5. doi:10.1142/S0218810411005588.

Johnston P, Getgood A, Larson D, Chojnowski AJ, Chakrabarti AJ, Chapman PG. De la Caffiniere thumb trapeziometacarpal joint arthroplasty: 16-26 year follow-up. *J Hand Surg Eur Vol*. Sep 2012;37(7):621-4. doi:10.1177/1753193411433226.

Kennedy AM, Barker J, Estfan R, Packer GJ. The use of the RegJoint implant for base of thumb osteoarthritis: Results with a minimum follow-up of 2 years. *Hand Surg Rehabil*. Feb 2020;39(1):53-58. doi:10.1016/j.hansur.2019.11.001.

Kessler FB, Epstein MJ, Culver JE, Prewitt J, Homsy CA. Proplast stabilized stemless trapezium implant. *The Journal of Hand Surgery*. 1984/03 1984;9(2):227-231. doi:10.1016/s0363-5023(84)80147-1

Kirkeby L, Frost P, Svendsen SW, Hansen TB. Revision rates of trapeziometacarpal total joint arthroplasty in relation to occupational hand force requirements. *J Hand Surg Eur Vol*. Nov 2021;46(9):968-974. doi:10.1177/1753193421996980

Klahn A, Nygaard M, Gvozdenovic R, Boeckstyns ME. Elektra prosthesis for trapeziometacarpal osteoarthritis: a follow-up of 39 consecutive cases. *J Hand Surg Eur Vol*. Sep 2012;37(7):605-9. doi:10.1177/1753193412443501

Kokkalis ZT, Zanaros G, Weiser RW, Sotereanos DG. Trapezium resection with suspension and interposition arthroplasty using acellular dermal allograft for thumb carpometacarpal arthritis. *J Hand Surg Am*. Jul-Aug 2009;34(6):1029-36. doi:10.1016/j.jhsa.2009.03.001

Kollig E, Weber W, Bieler D, Franke A. Failure of an uncemented thumb carpometacarpal joint ceramic prosthesis. *J Hand Surg Eur Vol*. Jul 2017;42(6):599-604. doi:10.1177/1753193416688427

Lallemand B, Cheval D, Camps C, Merle M, Jager T. Outcome of Silicone Implant for Treatment After Failure of Primary Trapeziometacarpal Surgery. *J Hand Surg Am*. May 2020;45(5):451 e1-451 e5. doi:10.1016/j.jhsa.2019.09.013

Lanzetta M, Foucher GJTJoHSB, Volume E. A comparison of different surgical techniques in treating degenerative arthrosis of the carpometacarpal joint of the thumb: a retrospective study of 98 cases. 1995;20(1):105-110.

Lehmann O, Herren D, Simmen B. Comparison of tendon suspension-interposition and silicon spacers in the treatment of degenerative osteoarthritis of the base of the thumb. Elsevier; 1998:25-30.

Lemoine S, Wavreille G, Alnot JY, Fontaine C, Chantelot C, groupe G. Second generation GUEPAR total arthroplasty of the thumb basal joint: 50 months follow-up in 84 cases. *Orthop Traumatol Surg Res*. Feb 2009;95(1):63-9. doi:10.1016/j.otsr.2008.06.001

Lister GD, Kleinert HE, Kutz JE, Atasoy EJH. Arthritis of the trapezial articulations treated by prosthetic replacement. 1977;(2):117-129.

Logan J, Peters SE, Strauss R, Manzanero S, Couzens GB, Ross M. Pyrocardan Trapeziometacarpal Joint Arthroplasty-Medium-Term Outcomes. *J Wrist Surg*. Dec 2020;9(6):509-517. doi:10.1055/s-0040-1714685

Logli AL, Twu J, Bear BJ, Lindquist JR, Schoenfeldt TL, Korcek KJ. Arthroscopic Partial Trapeziectomy With Soft Tissue Interposition for Symptomatic Trapeziometacarpal Arthritis: 6-Month and 5-Year Minimum Follow-Up. *J Hand Surg Am*. Apr 2018;43(4):384 e1-384 e7. doi:10.1016/j.jhsa.2017.10.016

Lovell M, Nuttall D, Trail I, Stilwell J, Stanley JJJoHS. A patient-reported comparison of trapeziectomy with Swanson Silastic implant or sling ligament reconstruction. 1999;24(4):453-455.

Lussiez B, Falaise C, Ledoux P. Dual mobility trapeziometacarpal prosthesis: a prospective study of 107 cases with a follow-up of more than 3 years. *J Hand Surg Eur Vol*. Nov 2021;46(9):961-967. doi:10.1177/17531934211024500

MacDermid JC, Roth JH, Rampersaud YR, Bain GIJCjos. Trapezial arthroplasty with silicone rubber implantation for advanced osteoarthritis of the trapeziometacarpal joint of the thumb. 2003;46(2):103.

Mariconda M, Russo S, Smeraglia F, Busco G. Partial trapeziectomy and pyrocarbon interpositional arthroplasty for trapeziometacarpal joint osteoarthritis: results after minimum 2 years of follow-up. *J Hand Surg Eur Vol*. Jul 2014;39(6):604-10. doi:10.1177/1753193413519384

Marks M, Hensler S, Wehrli M, Scheibler AG, Schindele S, Herren DB. Trapeziectomy With Suspension-Interposition Arthroplasty for Thumb Carpometacarpal Osteoarthritis: A Randomized Controlled Trial Comparing the Use of Allograft Versus Flexor Carpi Radialis Tendon. *J Hand Surg Am*. Dec 2017;42(12):978-986. doi:10.1016/j.jhsa.2017.07.023

Martinez de Aragon JS, Moran SL, Rizzo M, Reggin KB, Beckenbaugh RD. Early outcomes of pyrolytic carbon hemiarthroplasty for the treatment of trapezial-metacarpal arthritis. *J Hand Surg Am*. Feb 2009;34(2):205-12. doi:10.1016/j.jhsa.2008.10.018

Martin-Ferrero M, Simon-Perez C, Coco-Martin MB, Vega-Castrillo A, Aguado-Hernandez H, Mayo-Iscar A. Trapeziometacarpal total joint arthroplasty for osteoarthritis: 199 patients with a minimum of 10 years follow-up. *J Hand Surg Eur Vol*. Jun 2020;45(5):443-451. doi:10.1177/1753193419871660

Martin-Ferrero M. Ten-year long-term results of total joint arthroplasties with ARPE(R) implant in the treatment of trapeziometacarpal osteoarthritis. *J Hand Surg Eur Vol*. Oct 2014;39(8):826-32. doi:10.1177/1753193413516244

Martin-Ferrero MA, Trigueros-Larrea JM, Martin-de la Cal E, Coco-Martin B, Simon-Perez C. Long-Term Results of Joint Arthroplasty with Total Prosthesis for Trapeziometacarpal Osteoarthritis in Patients over 65 Years of Age. *Geriatrics (Basel)*. Jun 29 2021;6(3)doi:10.3390/geriatrics6030065

Martins A, Charbonnel S, Lecomte F, Athlani L. The Moovis(R) implant for trapeziometacarpal osteoarthritis: results after 2 to 6 years. *J Hand Surg Eur Vol*. Jun 2020;45(5):477-482. doi:10.1177/1753193420901435

Mattila S, Ainola M, Waris E. Bioabsorbable poly-L/D-lactide (96/4) scaffold arthroplasty (RegJoint) for trapeziometacarpal osteoarthritis: a 3-year follow-up study. *J Hand Surg Eur Vol*. May 2018;43(4):413-419. doi:10.1177/1753193417732002

Mattila S, Haapamaki V, Waris E. Unfavourable early outcomes of total trapeziectomy with RegJoint interposition: a report of 38 osteoarthritic hands in 34 patients. *J Hand Surg Eur Vol*. Feb 2020;45(2):167-172. doi:10.1177/1753193419885272

Mattila S, Waris E. Outcomes of Revision of Interposition Implant Trapeziometacarpal Arthroplasty. *Hand (N Y)*. Jul 23 2021:15589447211028920. doi:10.1177/15589447211028920

Mosegaard SB, Stilling M, Hansen TB. Risk factors for limited improvement after total trapeziometacarpal joint arthroplasty. *Health Qual Life Outcomes*. Mar 30 2020;18(1):90. doi:10.1186/s12955-020-01333-z

Naidu SH, Kulkarni N, Saunders M. Titanium basal joint arthroplasty: a finite element analysis and clinical study. *J Hand Surg Am*. May-Jun 2006;31(5):760-5. doi:10.1016/j.jhsa.2005.12.022

Nilsson A, Wiig M, Alnehill H, et al. The Artelon CMC spacer compared with tendon interposition arthroplasty. *Acta Orthop*. Apr 2010;81(2):237-44. doi:10.3109/17453671003635835

Nusem I, Goodwin DR. Excision of the Trapezium and Interposition Arthroplasty with Gelfoam for the Treatment of Trapeziometacarpal Osteoarthritis. *Journal of Hand Surgery*. 2016;28(3):242-245. doi:10.1016/s0266-7681(03)00009-3

Odella S, Querenghi AM, Sartore R, A DEF, Dacatra U. Trapeziometacarpal osteoarthritis: pyrocarbon interposition implants. *Joints*. Oct-Dec 2014;2(4):154-8.

Oh WT, Chun YM, Koh IH, Shin JK, Choi YR, Kang HJ. Tendon versus Pyrocarbon Interpositional Arthroplasty in the Treatment of Trapeziometacarpal Osteoarthritis. *Biomed Res Int*. 2019;2019:7961507. doi:10.1155/2019/7961507

O'Leary ST, Grobbelaar AO, Goldsmith N, Smith PJ, Harrison DH. Silicone arthroplasty for trapeziometacarpal arthritis. *J Hand Surg Br*. Oct 2002;27(5):457-61. doi:10.1054/jhsb.2002.0827.

Pendse A, Nisar A, Shah SZ, Bhosale A, Freeman JV, Chakrabarti I. Surface replacement trapeziometacarpal joint arthroplasty--early results. *J Hand Surg Eur Vol*. Dec 2009;34(6):748-57. doi:10.1177/1753193409343750.

Pritchett JW, Habryl LS. A promising thumb Basal joint hemiarthroplasty for treatment of trapeziometacarpal osteoarthritis. *Clin Orthop Relat Res*. Oct 2012;470(10):2756-63. doi:10.1007/s11999-012-2367-7

Regnard PJ. Electra trapezio metacarpal prosthesis: results of the first 100 cases. *J Hand Surg Br*. Dec 2006;31(6):621-8. doi:10.1016/j.jhsb.2006.05.019

Robles-Molina MJ, Lopez-Caba F, Gomez-Sanchez RC, Cardenas-Grande E, Pajares-Lopez M, Hernandez-Cortes P. Trapeziectomy With Ligament Reconstruction and Tendon Interposition Versus a Trapeziometacarpal Prosthesis for the Treatment of Thumb Basal Joint Osteoarthritis. *Orthopedics*. Jul 1 2017;40(4):e681-e686. doi:10.3928/01477447-20170503-03

Russo S, Bernasconi A, Busco G, Sadile F. Treatment of the trapeziometacarpal osteoarthritis by arthroplasty with a pyrocarbon implant. *Int Orthop*. Jul 2016;40(7):1465-71. doi:10.1007/s00264-015-3016-z

Sander AL, Buhrmann CF, Sommer K, Frank J. Simplified abductor pollicis longus suspension interposition arthroplasty for thumb carpometacarpal joint osteoarthritis. *Eur J Trauma Emerg Surg*. Dec 24 2020;doi:10.1007/s00068-020-01577-w

Semere A, Vuillerme N, Corcella D, Forli A, Moutet F. Results with the Roseland((R)) HAC trapeziometacarpal prosthesis after more than 10 years. *Chir Main*. Apr 2015;34(2):59-66. doi:10.1016/j.main.2015.01.004

Smeraglia F, Barrera-Ochoa S, Mendez-Sanchez G, Basso MA, Balato G, Mir-Bullo X. Partial trapeziectomy and pyrocarbon interpositional arthroplasty for trapeziometacarpal osteoarthritis: minimum 8-year follow-up. *J Hand Surg Eur Vol*. Jun 2020;45(5):472-476. doi:10.1177/1753193420906805

Sollerman C, Hasselgren G, Westermark J, Herrlin K. Replacement of the os trapezium by polyurethane implants. *Scand J Plast Reconstr Surg Hand Surg*. Sep 1993;27(3):217-21. doi:10.3109/02844319309078114

Sollerman C, Herrlin K, Abrahamsson S, Lindholm AJTJoHSB, Volume E. Silastic replacement of the trapezium for arthrosis—a twelve year follow-up study. 1988;13(4):426-429.

Søndergaard L, Konradsen L, Rechnagel K. Long-term follow-up of the cemented Caffinière prosthesis for trapezio-metacarpal arthroplasty. J Hand Surg Br. Nov 1991;16(4):428-30. doi:10.1016/0266-7681(91)90019-k

Sotereanos DG, Taras J, Urbaniak JRJTJohs. Niebauer trapeziometacarpal arthroplasty: a long-term follow-up. 1993;18(4):560-564.

Spaans AJ, van Heeswijk EJ, Arnold DE, Beumer A. Foreign body reaction associated with polyethylene mesh interposition used for treatment of trapeziometacarpal osteoarthritis: report of 8 cases. *J Hand Surg Am*. Oct 2014;39(10):2016-9. doi:10.1016/j.jhsa.2014.07.038

Spaans AJ, van Minnen LP, Weijns ME, Braakenburg A, van der Molen AB. Retrospective Study of a Series of 20 Ivory Prostheses in the Treatment of Trapeziometacarpal Osteoarthritis. *J Wrist Surg*. May 2016;5(2):131-6. doi:10.1055/s-0036-1571283

Stillwater L, Memauri B, Ratanshi I, Islur A, Amaratunga T. Radiographic interpretation of carpometacarpal arthroplasty: correlation between radiographic loosening and clinical outcome. *Skeletal Radiol*. Aug 2017;46(8):1057-1062. doi:10.1007/s00256-017-2648-z

Swanson AB, Swanson Gd, Watermeier JJ. Trapezium implant arthroplasty. *The Journal of Hand Surgery*. 1981/03 1981;6(2):125-141. doi:10.1016/s0363-5023(81)80165-7

Szalay G, Meyer C, Scheufens T, Schnettler R, Christ R, Schleicher IJAOB. Pyrocarbon spacer as a trapezium replacement for arthritis of the trapeziometacarpal joint; a follow-up study of 60 cases. 2013;79(6):648-54.

Taylor EJ, Desari K, D'Arcy JC, Bonnici AV. A comparison of fusion, trapeziectomy and silastic replacement for the treatment of osteoarthritis of the trapeziometacarpal joint. *J Hand Surg Br*. Feb 2005;30(1):45-9. doi:10.1016/j.jhsb.2004.08.006

Tchurukdichian A, Gerenton B, Moris V, See LA, Stivala A, Guillier D. Outcomes of Double-Mobility Prosthesis in Trapeziometacarpal Joint Arthritis With a Minimal 3 Years of Follow-Up: An Advantage for Implant Stability. *Hand (N Y)*. May 2021;16(3):368-374. doi:10.1177/1558944719855690

Tchurukdichian A, Guillier D, Moris V, See LA, Macheboeuf Y. Results of 110 IVORY(R) prostheses for trapeziometacarpal osteoarthritis with a minimum follow-up of 10 years. *J Hand Surg Eur Vol*. Jun 2020;45(5):458-464. doi:10.1177/1753193419899843

Thillemann JK, Thillemann TM, Munk B, Kroner K. High revision rates with the metal-on-metal Motec carpometacarpal joint prosthesis. *J Hand Surg Eur Vol*. Mar 2016;41(3):322-7. doi:10.1177/1753193415595527

Toffoli A, Teissier J. MAIA Trapeziometacarpal Joint Arthroplasty: Clinical and Radiological Outcomes of 80 Patients With More than 6 Years of Follow-Up. *J Hand Surg Am*. Oct 2017;42(10):838 e1-838 e8. doi:10.1016/j.jhsa.2017.06.008.

Ulrich-Vinther M, Puggaard H, Lange B. Prospective 1-year follow-up study comparing joint prosthesis with tendon interposition arthroplasty in treatment of trapeziometacarpal osteoarthritis. *J Hand Surg Am*. Oct 2008;33(8):1369-77. doi:10.1016/j.jhsa.2008.04.028

van Aaken J, Holzer N, Wehrli L, Delaquaize F, Gonzalez IA, Beaulieu JY. Unacceptable failure of the PI2(R) implant. *J Hand Surg Eur Vol*. Nov 2016;41(9):917-922. doi:10.1177/1753193416651573

Van Cappelle HG, Deutman R, Van Horn JRJJ. Use of the Swanson silicone trapezium implant for treatment of primary osteoarthritis: long-term results. 2001;83(7):999-1004.

van Cappelle HG, Elzenga P, van Horn JRJTJohs. Long-term results and loosening analysis of de la Caffiniere replacements of the trapeziometacarpal joint. 1999;24(3):476-482.

van Laarhoven C, Ottenhoff JSE, van Hoorn B, van Heijl M, Schuurman AH, van der Heijden B. Medium to Long-Term Follow-Up After Pyrocarbon Disc Interposition Arthroplasty for Treatment of CMC Thumb Joint Arthritis. *J Hand Surg Am*. Feb 2021;46(2):150 e1-150 e14. doi:10.1016/j.jhsa.2020.07.025

Vissers G, Goorens CK, Vanmierlo B, et al. Ivory arthroplasty for trapeziometacarpal osteoarthritis: 10-year follow-up. *J Hand Surg Eur Vol*. Feb 2019;44(2):138-145. doi:10.1177/1753193418797890

Wachtl S, Guggenheim P, Sennwald GJTJob, volume jsB. Cemented and non-cemented replacements of the trapeziometacarpal joint. 1998;80(1):121-125.

Zollinger PE, Ellis ML, Unal H, Tuinebreijer WE. Clinical outcome of cementless semi-constrained trapeziometacarpal arthroplasty, and possible effect of vitamin C on the occurrence of complex regional pain syndrome. *Acta orthopaedica Belgica*. Jun 2008;74(3):317-22.
