# Supplementary figures and images for: Efficacy and Safety of Different Trapezium Implants for Trapeziometacarpal Joint Osteoarthritis: A Systematic Review and Meta-Analysis
Source: Hand (N Y). 2023 Jul 2;19(8):1242–51. doi: 10.1177/15589447231183172 (PMC11612267; doi:10.1177/15589447231183172)

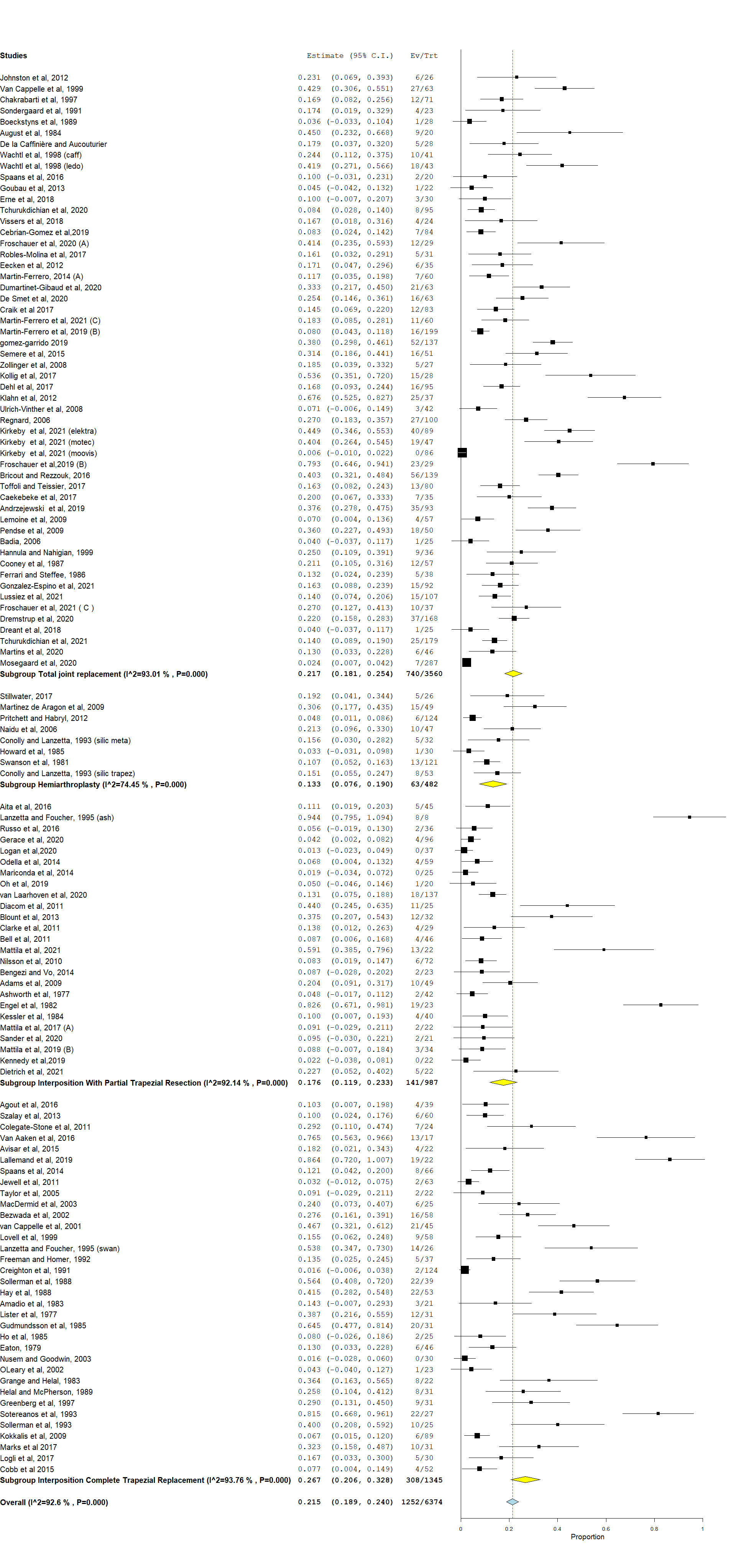

Supplement: sj-tiff-10-han-10.1177_15589447231183172 – Supplemental material for Efficacy and Safety of Different Trapezium Implants for Trapeziometacarpal Joint Osteoarthritis: A Systematic Review and Meta-Analysis [file sj-tiff-10-han-10.1177_15589447231183172.tiff]

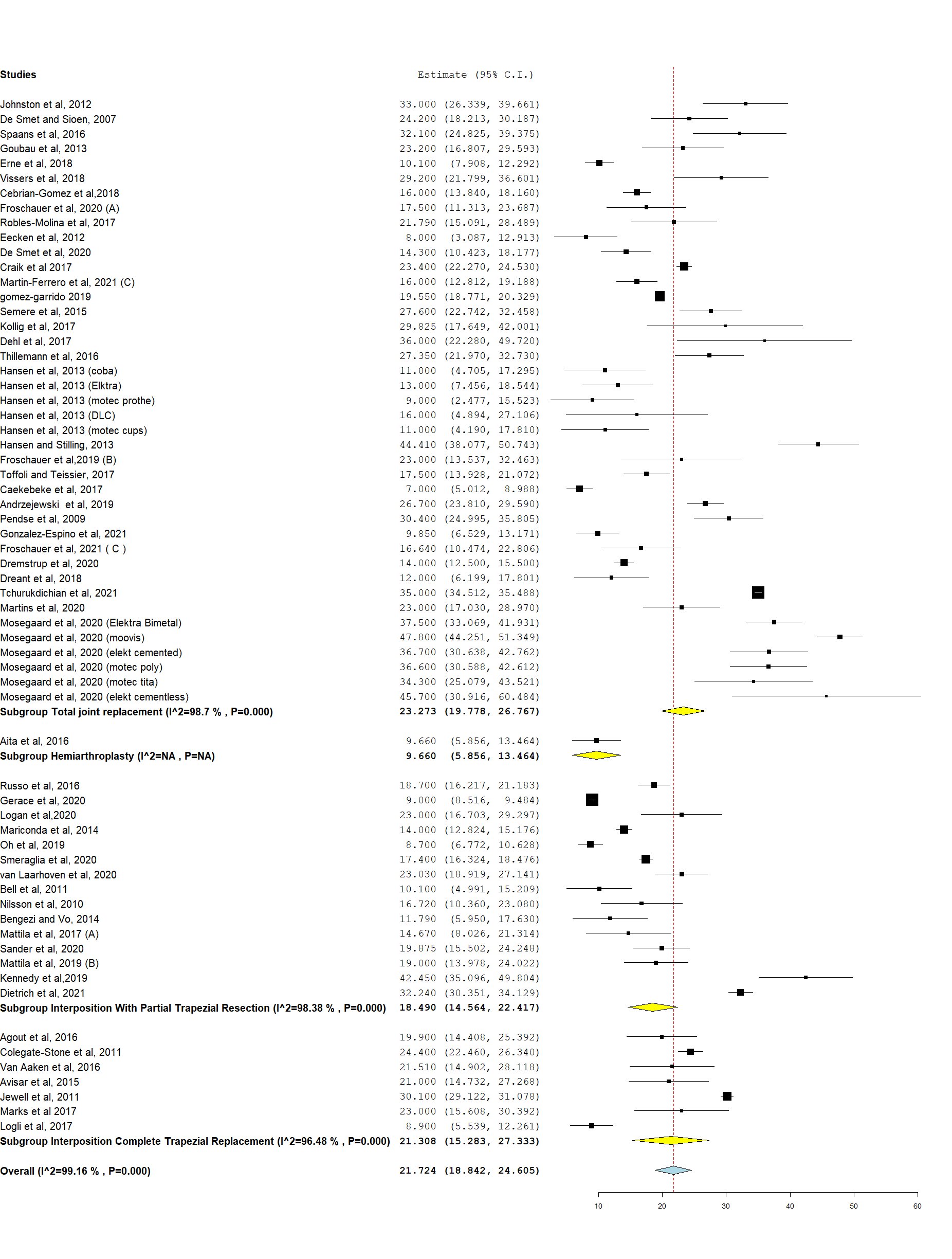

Supplement: sj-tiff-7-han-10.1177_15589447231183172 – Supplemental material for Efficacy and Safety of Different Trapezium Implants for Trapeziometacarpal Joint Osteoarthritis: A Systematic Review and Meta-Analysis [file sj-tiff-7-han-10.1177_15589447231183172.tiff]

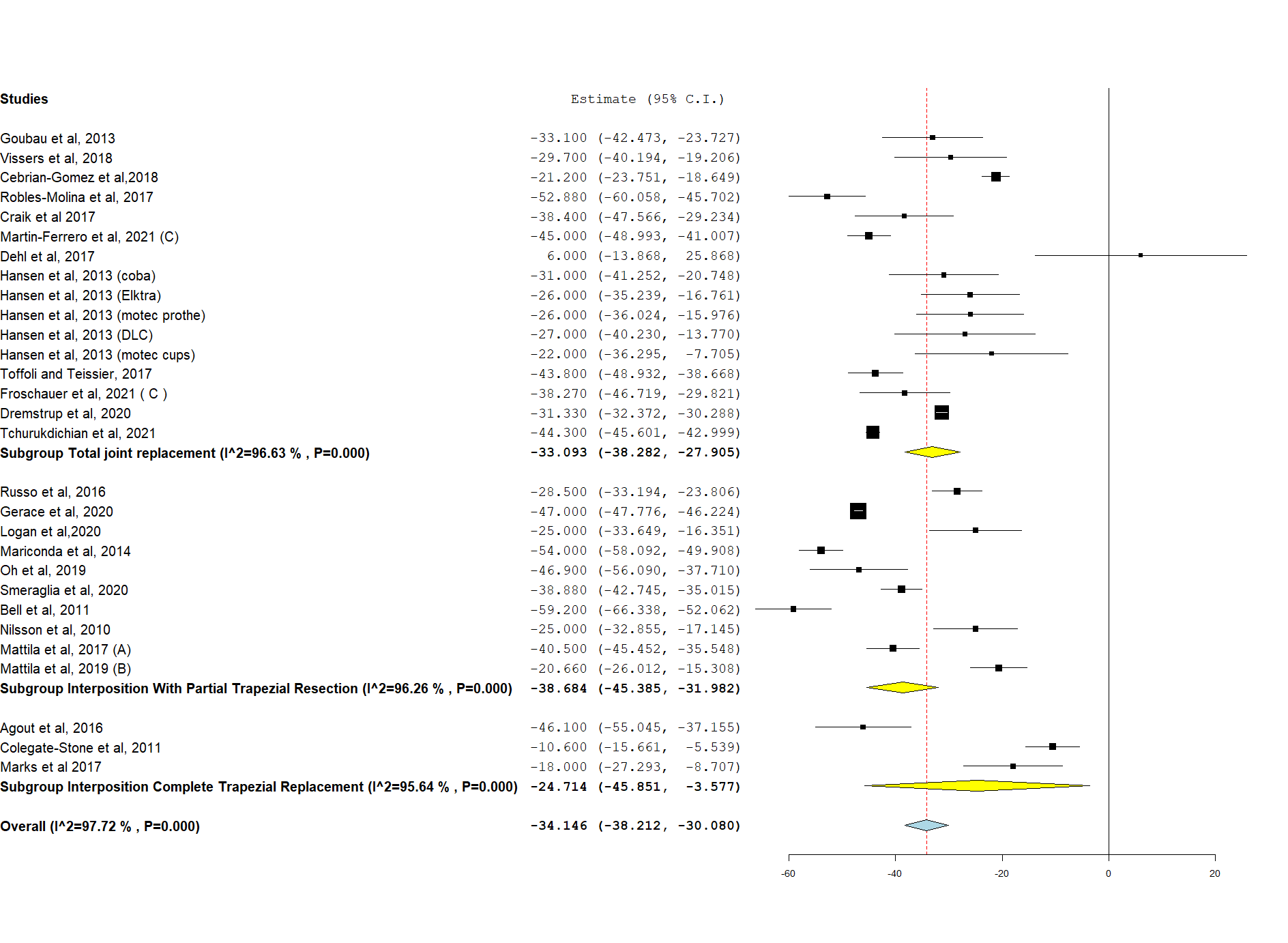

Supplement: sj-tiff-8-han-10.1177_15589447231183172 – Supplemental material for Efficacy and Safety of Different Trapezium Implants for Trapeziometacarpal Joint Osteoarthritis: A Systematic Review and Meta-Analysis [file sj-tiff-8-han-10.1177_15589447231183172.tiff]

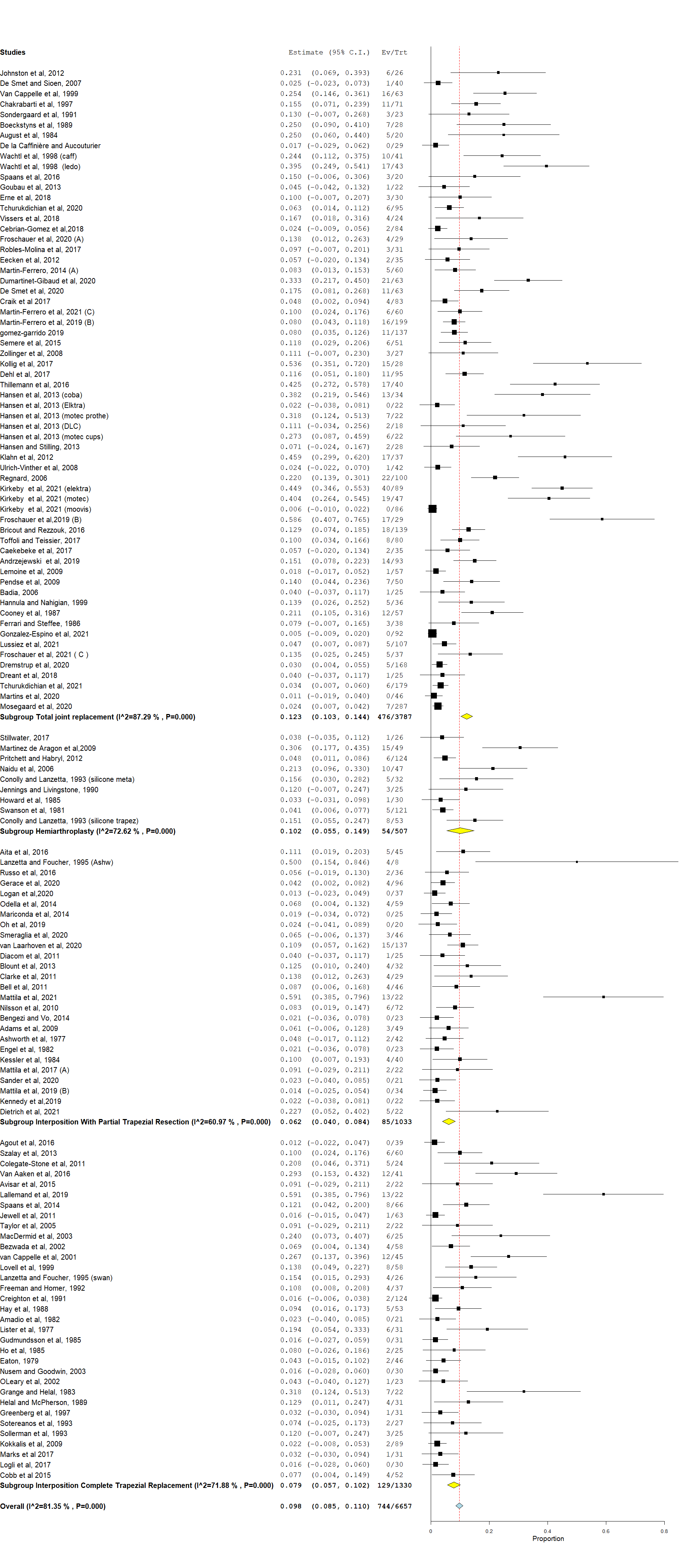

Supplement: sj-tiff-9-han-10.1177_15589447231183172 – Supplemental material for Efficacy and Safety of Different Trapezium Implants for Trapeziometacarpal Joint Osteoarthritis: A Systematic Review and Meta-Analysis [file sj-tiff-9-han-10.1177_15589447231183172.tiff]
